# The *Aspergillus fumigatus* CrzA Transcription Factor Activates Chitin Synthase Gene Expression during the Caspofungin Paradoxical Effect

**DOI:** 10.1128/mBio.00705-17

**Published:** 2017-06-13

**Authors:** Laure Nicolas Annick Ries, Marina Campos Rocha, Patrícia Alves de Castro, Rafael Silva-Rocha, Roberto Nascimento Silva, Fernanda Zanolli Freitas, Leandro José de Assis, Maria Célia Bertolini, Iran Malavazi, Gustavo H. Goldman

**Affiliations:** a Faculdade de Ciências Farmacêuticas de Ribeirão Preto, Universidade de São Paulo, Ribeirão Preto, São Paulo, Brazil; b Departamento de Genética e Evolução, Centro de Ciências Biológicas e da Saúde, Universidade Federal de São Carlos, São Carlos, São Paulo, Brazil; c Faculdade de Medicina de Ribeirão Preto, Universidade de São Paulo, Ribeirão Preto, São Paulo, Brazil; d Universidade Estadual Paulista, UNESP, Instituto de Química, Araraquara, São Paulo, Brazil; Duke University Medical Center

**Keywords:** *Aspergillus fumigatus*, cell wall integrity pathway, caspofungin, paradoxical effect

## Abstract

*Aspergillus fumigatus* is an opportunistic fungal pathogen that causes invasive aspergillosis (IA), a life-threatening disease in immunocompromised humans. The echinocandin caspofungin, adopted as a second-line therapy in combating IA, is a β-1,3-glucan synthase inhibitor, which, when used in high concentrations, reverts the anticipated *A. fumigatus* growth inhibition, a phenomenon called the “caspofungin paradoxical effect” (CPE). The CPE has been widely associated with increased chitin content in the cell wall due to a compensatory upregulation of chitin synthase-encoding genes. Here, we demonstrate that the CPE is dependent on the cell wall integrity (CWI) mitogen-activated protein kinase MpkA^MPK1^ and its associated transcription factor (TF) RlmA^RLM1^, which regulate chitin synthase gene expression in response to different concentrations of caspofungin. Furthermore, the calcium- and calcineurin-dependent TF CrzA binds to and regulates the expression of specific chitin synthase genes during the CPE. These results suggest that the regulation of cell wall biosynthetic genes occurs by several cellular signaling pathways. In addition, CrzA is also involved in cell wall organization in the absence of caspofungin. Differences in the CPE were also observed between two *A. fumigatus* clinical isolates, which led to the identification of a novel basic leucine zipper TF, termed ZipD. This TF functions in the calcium-calcineurin pathway and is involved in the regulation of cell wall biosynthesis genes. This study therefore unraveled additional mechanisms and novel factors governing the CPE response, which ultimately could aid in developing more effective antifungal therapies.

## IMPORTANCE

Systemic *Aspergillus fumigatus* infections are often accompanied by high mortality rates. The fungal cell wall is important for infection as it has immunomodulatory and immunoevasive properties. Paradoxical growth of *A. fumigatus* in the presence of high concentrations of the cell wall-disturbing agent caspofungin has been observed for more than a decade, although the mechanistic nature of this phenomenon remains largely uncharacterized. Here, we show that the CWI pathway components MpkA and RlmA as well as the calcium/calcineurin-responsive transcription factor CrzA regulate the expression of cell wall biosynthetic genes during the caspofungin paradoxical effect (CPE). Furthermore, an additional, novel calcium/calcineurin-responsive transcription factor was identified to play a role in cell wall biosynthesis gene expression during the CPE. This work paints a crucial role for calcium metabolism in the CPE and provides further insight into the complex regulation of cell wall biosynthesis, which could ultimately lead to the development of more efficient antifungal therapies.

## INTRODUCTION

Invasive aspergillosis (IA) has emerged as one of the most common life-threatening fungal diseases in immunocompromised humans, and mortality rates as high as 90% have been reported (1–5). Systemic fungal infections such as IA are usually treated with antifungal drugs such as polyenes, azoles, and echinocandins, with the first two targeting cell membrane ergosterol biosynthesis and the latter perturbing the biosynthesis of the cell wall (CW) polysaccharide glucan (6). Echinocandins represent a relatively new class of antifungal agents, which act by noncompetitively inhibiting the cell wall enzyme β-1,3-glucan synthase, therefore impairing fungal cell wall biosynthesis and integrity (7). Echinocandins present fungistatic activity against *Aspergillus* spp. and are regularly used as second-line therapy for IA (8).

The fungal cell wall, mainly composed of glucan and chitin, mediates the interaction with human host cells, playing an important role in the evasion and modulation of the host immune system (9). Fungal cell wall plasticity is maintained by the cell wall integrity (CWI) pathway, a signaling cascade, conserved in yeast and filamentous fungi, which is activated in response to various stresses and which aims at protecting the cell wall in order to ensure survival of the fungus. In *Aspergillus fumigatus*, the CWI pathway encompasses a mitogen-activated protein kinase (MAPK) cascade, resulting in MpkA phosphorylation and relocalization to the nucleus, where it stimulates a transcriptional response (10–12). Deletion of *mpkA* results in increased stress to cell wall (CW)-disturbing agents as well as in impaired cell wall composition (10, 11). Recently, the MADS box transcription factor RlmA was identified as functioning downstream of and regulating the phosphorylation of MpkA, therefore contributing to the regulation of CWI (13).

It has been known for over a decade that high concentrations of the echinocandin caspofungin can revert the anticipated *A. fumigatus* growth inhibition, a phenomenon termed the “caspofungin paradoxical effect” (CPE) (1). In *A. fumigatus* and *Candida albicans*, the CPE is widely associated with increased chitin content in the cell wall due to a compensatory upregulation of chitin synthase-encoding genes (14–17). More recently, it was proposed that the *A. fumigatus* β-glucan synthase Fks1 is essential for maintaining the paradoxical effect at the later stages during growth on high concentrations of caspofungin (18). Furthermore, high concentrations of caspofungin elicit a spike in cytosolic Ca^2+^, and calcium metabolism has been shown to be important for the subsistence of the CPE, through the activation of calcineurin and its associated major transcription factor CrzA (19, 20). Indeed, phosphorylation of calcineurin was increased in paradoxical growth concentrations of caspofungin (20). In addition, calcineurin was shown to be involved in the regulation of chitin synthase gene expression, providing a link between CWI and calcium signaling (19). Similarly, the heat shock chaperone Hsp90 is also involved in the CPE, with modifications in the *hsp90* promoter region or geldanamycin-induced inhibition of Hsp90 resulting in the loss of the CPE in *A. fumigatus* (21, 22).

Despite the abovementioned studies, the details of the mechanistic nature of the CPE in *A. fumigatus* remain unknown, although they can provide an opportunity to identify genetic elements regulating cell wall biosynthesis. This study therefore set out to unravel the molecular events governing the CPE in *A. fumigatus*. The CPE was shown to be dependent on the MAPK MpkA^MPK1^ and the transcription factor (TF) RlmA^RLM1^, both of which regulate the expression of chitin synthase-encoding genes in the presence of different caspofungin concentrations. Furthermore, the calcium- and calcineurin-dependent TF CrzA was shown to bind to the promoter regions and regulate the expression of specific chitin synthase genes during the CPE. These results suggest that chitin synthase genes are regulated by different cellular pathways. Differences in the CPE were observed between two *A. fumigatus* clinical isolates, which led to the identification of the basic leucine zipper TF ZipD, which also responds to calcium-calcineurin signaling and is involved in the regulation of chitin biosynthesis genes during the CPE. In summary, this study provides new insights into cell wall biosynthesis regulation, which could potentially lead to more efficient antifungal therapies for treatment of IA.

## RESULTS

### The MpkA-RlmA-CWI pathway is involved in the CPE response.

**FIG 1:**
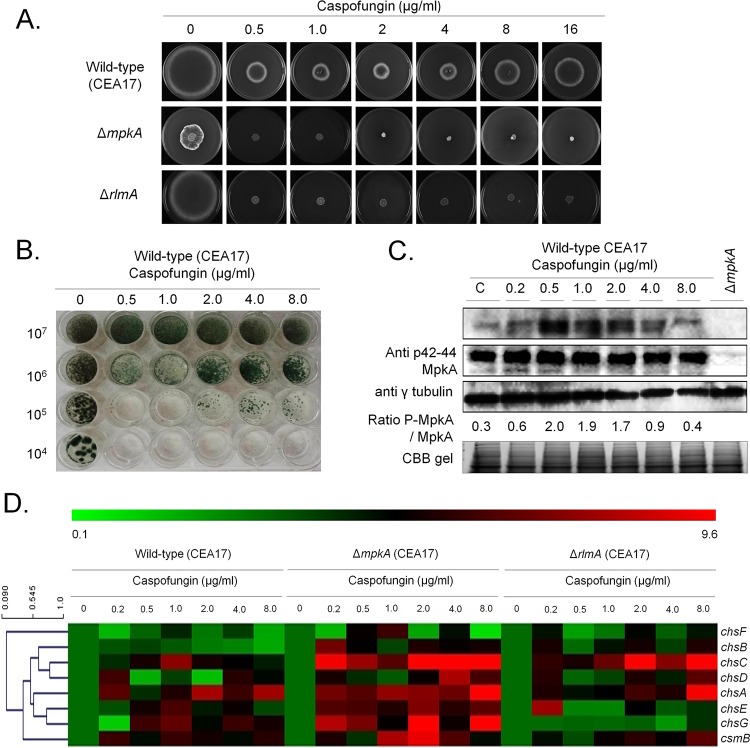
The MpkA-CWI integrity pathway is activated during the CPE. (A) *A. fumigatus* CEA17 conidia (10^4^) were inoculated on solid minimal medium (MM) supplemented with glucose and different caspofungin concentrations for 5 days at 37°C. (B) Different concentrations of *A. fumigatus* CEA17 conidia (left) were inoculated in liquid MM supplemented with glucose and different caspofungin concentrations for 16 h at 37°C. (C) Western blotting assay of MpkA phosphorylation in response to increasing caspofungin concentrations. Anti-p44/42 MpkA or anti-44/42 MpkA antibodies were used to detect the phosphorylation of MpkA and total MpkA, respectively. Anti-γ-tubulin antibody was used as a loading control. Signal intensities were quantified using the ImageJ software, and ratios of (P)-MpkA to MpkA were calculated. A Coomassie brilliant blue (CBB) gel of the protein extract served as an additional loading control. (D) Heat map and hierarchical linkage clustering (as determined by MeV software) of RT-qPCRs of the chitin synthase gene mRNA accumulation in the presence of caspofungin in the wild-type, Δ*mpkA*, and Δ*rlmA* strains. Strains were grown for 16 h at 37°C and transferred to increasing caspofungin concentrations for 60 min. The results are expressed as the average of the fold increase of the control cDNA (without caspofungin) for a specific gene of three biological independent experiments (with 2 technical repetitions each; see Fig. S1).

Considering that caspofungin targets cell wall glucan biosynthesis, a role of the cell wall integrity (CWI) pathway in the CPE was investigated. Deletion of the CWI MAPK gene *mpkA* and its associated TF gene *rlmA* resulted in the loss of the CPE when grown in the presence of increasing concentrations of caspofungin on solid medium (Fig. 1A). Fungal mycelia grown in liquid cultures are more sensitive to high concentrations of caspofungin, and in agreement, the CPE of *A. fumigatus* strain CEA17 was already observed at 2 μg/ml in liquid minimal medium (MM) (Fig. 1B), whereas on solid medium the onset of the CPE was observed at 8 μg/ml (Fig. 1A). In order to confirm the activation of the CWI during the CPE, MpkA phosphorylation was assessed by Western blotting in strain CEA17 when grown in complete medium (CM) and after the addition of caspofungin to 0.2, 0.5, 1.0, 2.0, 4.0, and 8.0 μg/ml for 1 h. Increased MpkA phosphorylation was observed in low caspofungin concentrations (2- to 6-fold increase in 0.2 to 1.0 µg/ml caspofungin, respectively) and in lower CPE concentrations (6- to 3-fold increase in 2 and 4 µg/ml caspofungin, respectively), whereas the phosphorylation of MpkA at 8 µg/ml caspofungin was similar to that under the control condition (Fig. 1C).

To determine whether the CPE was accompanied by an increase in chitin synthesis, the expression of the eight *A. fumigatus* chitin synthase-encoding genes was determined. Results show an increase in mRNA accumulation of the four chitin synthase-encoding genes *chsA*, *chsC*, *chsG*, and *csmB* when the wild-type strain was exposed to increasing concentrations (0.2 to 8 µg/ml) of caspofungin, suggesting a compensatory increase in chitin synthesis in response to caspofungin (Fig. 1D; see also Fig. S1A in the supplemental material). To determine whether MpkA and its downstream transcription factor RlmA are involved in controlling chitin synthase gene expression during the CPE, reverse transcription-quantitative PCR (RT-qPCR) of these genes in the Δ*mpkA* and Δ*rlmA* strains was performed (Fig. 1D; Fig. S1B and C). Deletion of *mpkA* resulted in increased expression of *chsA*, -*B*, -*C*, -*D*, -*E*, and -*G* and *csmB* in the presence of high caspofungin concentrations, suggesting that MpkA exerts a repressive regulation on these genes. RlmA, on the other hand, seems to be important for the expression of *chsE* to -*G* but, similarly to MpkA, is involved in the repression of *chsC* (Fig. 1D).

10.1128/mBio.00705-17.1 FIG S1  Chitin synthase gene expression, as determined by RT-qPCR, in the wild-type (WT) (A), Δ*mpkA* (B), Δ*rlmA* (C), Δ*crzA* (D), and Δ*zipD* (E) strains when grown for 16 h at 37°C in minimal medium supplemented with glucose and after the addition of increasing caspofungin concentrations for 60 min. Standard deviations present the average from 3 independent biological repetitions (each with 2 technical repetitions). Statistical analysis was performed using a one-way ANOVA with Dunnett’s *post hoc* test when compared to the control condition (*, *P* < 0.05). Download FIG S1, PDF file, 1.8 MB. Copyright © 2017 Ries et al. 2017 Ries et al. This content is distributed under the terms of the Creative Commons Attribution 4.0 International license.

Taken together, these results indicate that caspofungin, at lower CPE concentrations, activates the CWI pathway but MpkA does not seem to be required for the response to higher CPE concentrations. The CPE is accompanied by an increase in chitin synthase expression which appears to be dependent on RlmA and not MpkA, suggesting CPE MpkA-independent functions of RlmA. However, MpkA seems to modulate transcriptionally the chitin synthase expression.

### CrzA binds to the promoter regions of certain chitin synthase-encoding genes.

**FIG 2:**
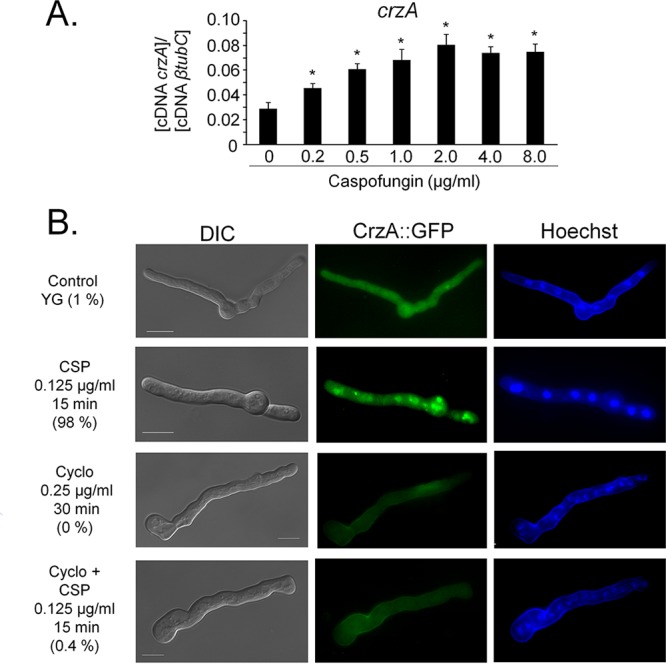
CrzA translocates to the nucleus upon caspofungin exposure. (A) The expression of *crzA*, as determined by RT-qPCR, is induced in the presence of caspofungin. The wild-type strain was grown for 16 h at 37°C and transferred to increasing caspofungin concentrations for 60 min. All gene expression was normalized by the amount of β-tubulin (*tubC*). Standard deviations present the average from 3 independent biological repetitions (each with 2 technical repetitions). Statistical analysis was performed using a one-way ANOVA with Dunnett’s *post hoc* test compared to the control condition (*, *P* < 0.05). (B) Cellular localization of the CrzA::GFP strain, as determined by microscopy, when grown for 16 h at 30°C and after incubation in the presence of caspofungin (CSP), cyclosporine (Cyclo), or both cyclosporine and caspofungin. The percentage of CrzA::GFP nuclear localization is indicated for each condition and is based on counting between 300 and 600 nuclei in 50 to 100 hyphal germlings of biological triplicates. Bars, 5 μm.

To further investigate the processes governing the upregulation of the chitin synthase-encoding genes, the promoter regions of the eight chitin synthase-encoding genes were screened for potential TF binding sites using the MEME predictor (23) and the motif comparison tool TOM-TOM (24). Three of the eight predicted motifs (Table S1) were identified as binding sequences for several TFs, including Crz1p [5′-TCA(GT)CCAC-3′], the homologue of *A. fumigatus* CrzA, whereas the remaining five motifs are predicted to not be recognized by any known TFs. As calcium metabolism was shown to be important for the CPE (19, 20), a role of CrzA in the cellular response to high levels of caspofungin was further investigated. The transcriptional expression of *crzA* in the *A. fumigatus* strain was significantly induced upon increasing caspofungin concentrations (Fig. 2A), and CrzA::GFP (green fluorescent protein) localized to the nuclei (98%) when hyphal germlings were exposed to 0.125 µg/ml caspofungin for 15 min (note that lower concentrations of caspofungin were used because germlings are much more sensitive to caspofungin than mycelia [Fig. 2B]). Cellular localization of CrzA::GFP in the presence of caspofungin is dependent on calcineurin, as prior incubation of the germlings with the calcineurin inhibitor cyclosporine for 30 min abolished CrzA::GFP nuclear translocation (0.4%) (Fig. 2B). The CrzA::GFP strain presented the same growth phenotype as the wild-type strain in the absence and presence of high concentrations of calcium, indicating that CrzA::GFP is functional (Fig. S2A and B).

10.1128/mBio.00705-17.2 FIG S2  The CrzA::GFP and ZipD::GFP strains are functional. (A) The wild-type, *crzA* and *zipD* deletion, complemented, and GFP-tagged strains were grown from 10^5^ spores for 5 days on minimal medium (MM) or complete medium (YAG) in the presence and absence of 500 mM CaCl_2_. (B) Also shown are the graphical representations of the average from three biological replicates. For each strain, colony diameters of the calcium-treated plates were divided by the colony diameters of the control condition. Statistical analysis was performed using a one-way ANOVA when compared to the control condition (*, *P* < 0.05). (C) Heat map and hierarchical linkage clustering (as determined by the MeV software) of RT-qPCRs of the chitin synthase gene mRNA accumulation in the presence of caspofungin in the wild-type and Δ*crzA* strains. Strains were grown for 16 h at 37°C and transferred to increasing caspofungin concentrations for 60 min. The results are expressed as the average of the fold increase of the control cDNA (without caspofungin) for a specific gene from three biological independent experiments (with 2 technical repetitions each). Download FIG S2, PDF file, 0.5 MB. Copyright © 2017 Ries et al. 2017 Ries et al. This content is distributed under the terms of the Creative Commons Attribution 4.0 International license.

10.1128/mBio.00705-17.5 TABLE S1  Putative transcription factor binding motifs identified in the chitin synthase promoter regions when using the MEME predictor and the motif comparison tool TOM-TOM. Download TABLE S1, PDF file, 0.3 MB. Copyright © 2017 Ries et al. 2017 Ries et al. This content is distributed under the terms of the Creative Commons Attribution 4.0 International license.

**FIG 3:**
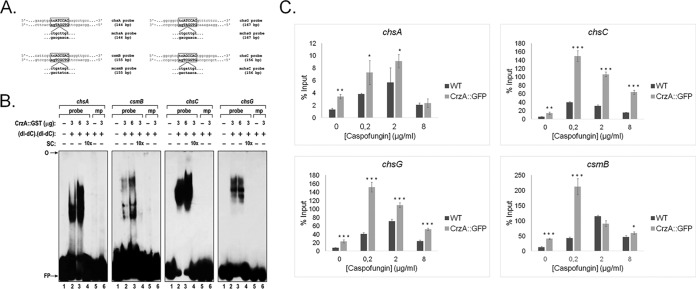
*A. fumigatus* CrzA transcription factor binds to the promoter regions of specific chitin synthase-encoding genes. (A) Schematic representation of the DNA probes (including mutated probes) of the *chsA*, *csmB*, *chsC*, and *chsG* promoter regions used during the electrophoretic mobility shift assays (EMSAs). (B) EMSAs using the GST::CrzA-tagged protein. Probes containing the endogenous or mutated CrzA motif were assayed for DNA-binding activity, using the recombinant GST::CrzA protein. The specificity of the DNA-protein binding was confirmed by adding specific competitors (cold DNA probes) and the mutated probes. SC, specific competitor; mp, mutated probes; FP, free probe. (C) ChIP-qPCR of the *chsA*, *csmB*, *chsC*, and *chsG* genes in the wild-type and CrzA::GFP strains when grown for 24 h in MM and then exposed to increasing concentrations of caspofungin for 1 h. Standard deviations present the average from two independent biological experiments (with 2 technical repetitions each). Statistical analysis was performed using a one-tailed, paired *t* test compared to the control condition (*, *P* < 0.05; **, *P* < 0.005; ***, *P* < 0.0005).

To confirm CrzA binding to the chitin synthase-encoding genes, electrophoretic mobility shift assays (EMSAs) and chromatin immunoprecipitation coupled to real-time PCR (ChIP-qPCR) were carried out. For the EMSAs, DNA fragments of approximately 200 bp, encompassing the CrzA calcineurin-dependent reporter element (CDRE) consensus binding sequence of the *chsA*, *chsC*, *chsG*, and *csmB* genes (Fig. 3A) were used as probes, and the recombinant glutathione *S*-transferase (GST)::CrzA (25, 26) was produced in *Escherichia coli* and purified. DNA-protein complexes with reduced mobility were observed for all four DNA fragments, indicating *in vitro* binding of CrzA to these DNA sequences (Fig. 3B). However, the affinities of the four DNA fragments to form complexes with various quantities (3 μg and 6 μg) of GST::CrzA were different, suggesting differing affinities of CrzA for the four gene promoter sequences (Fig. 3B). Site-directed mutagenesis of the DNA core sequence confirmed the specificity of the DNA-CrzA protein complexes. The addition of a 10-fold molar excess of unlabeled mutated probes (specific competitors [SCs]) to the labeled probes completely inhibited the formation of the DNA-protein complexes for all four DNA sequences (Fig. 3B). ChIP-PCR of the CrzA::GFP strain, when grown for 24 h in minimal medium supplemented with glucose and then transferred to 0.2, 2.0, and 8.0 µg/ml of caspofungin for 1 h, showed significant *in vivo* binding of CrzA to the promoter regions of *chsC* and *chsG* under all tested conditions and to the promoter regions of *chsA* and *csmB* in the presence of low concentrations of caspofungin compared to the negative (no-GFP) control strain (Fig. 3C). Taken together, these results indicate that *A. fumigatus* CrzA binds to and controls the expression of *chsA*, *chsC*, *chsG*, and *csmB* in the presence of various concentrations of caspofungin. In agreement, RT-qPCR of all chitin synthase-encoding genes in the Δ*crzA*^CEA17^ strain showed different expression patterns than those in the wild-type strain under the same conditions (Fig. S1D and S2C). In the Δ*crzA*^CEA17^ strain, highly increased levels of *chsD* and *csmB* compared to the wild-type strain in all tested caspofungin concentrations were observed, as well as additional minor caspofungin concentration-dependent differences in chitin synthase gene expression (Fig. S1D and S2C).

### CrzA is also involved in cell wall synthesis-related processes in the absence of caspofungin.

**FIG 4:**
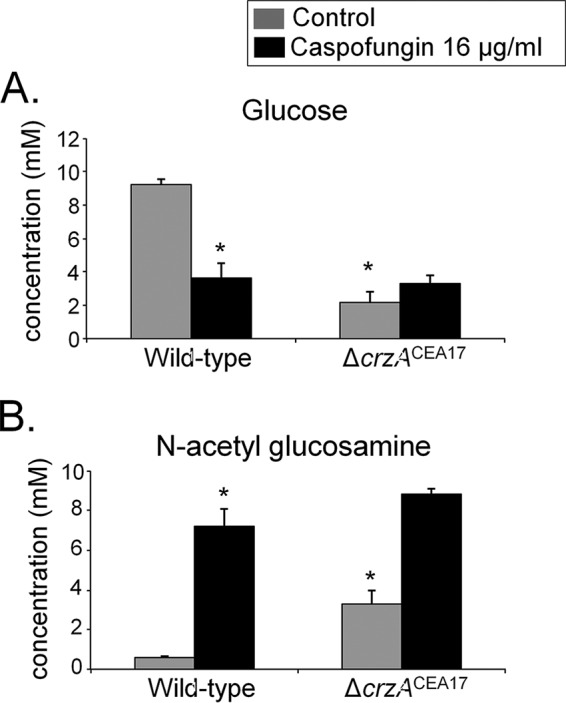
CrzA is important for cell wall composition in the absence of caspofungin. Glucose (A) and *N*-acetylglucosamine (NAG) (B) concentrations, as determined by high-performance liquid chromatography (HPLC), in mycelial extracts of the *A. fumigatus* wild-type (CEA17) and Δ*crzA*^CEA17^ strains when grown for 16 h in minimal medium (MM) at 37°C (control) and after a 1-h incubation in MM supplemented with 16 µg/ml caspofungin.

**FIG 5:**
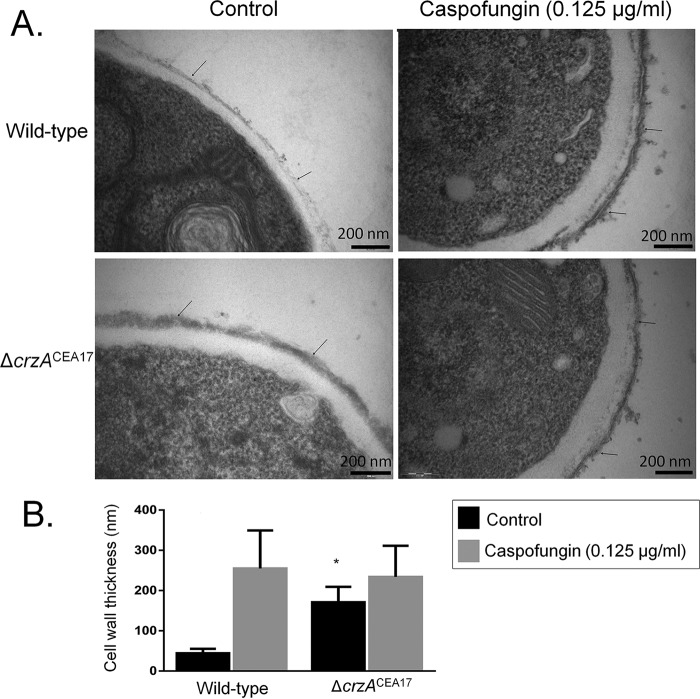
CrzA is involved in the cell wall integrity (CWI) response. (A) Transmission electron microscopy (TEM) of mycelial sections of the *A. fumigatus* wild-type (CEA17) and Δ*crzA*^CEA17^ strains when grown for 16 h in MM at 37°C (control) and after transfer to 0.125 µg/ml caspofungin for 2 h. Arrows indicate the external borders of the cell wall. (B) The cell wall thickness of 50 sections of different hyphal germlings (average of 4 sections per germling) was measured when grown under the same conditions as specified for panel A. Standard deviations present the average from the 50 measurements, and statistical analysis was performed using a one-tailed, paired *t* test compared to the control condition (*, *P* < 0.05).

To further study the role of CrzA in fungal CWI during the CPE, cell wall glucose and *N*-acetylglucosamine (NAG) contents were measured in the *A. fumigatus* CEA17 and Δ*crzA*^CEA17^ strains when grown for 16 h in minimal medium supplemented with glucose and then exposed to 16 μg/ml caspofungin for 1 h. In the wild-type strain, cell wall glucose concentrations decreased, whereas the NAG content increased upon incubation in caspofungin-rich medium (Fig. 4). The Δ*crzA*^CEA17^ strain showed amounts of glucose decreased from and comparable to those of the wild-type strain in the absence and presence of caspofungin (Fig. 4A). The NAG content, on the other hand, is about 3-fold higher in the Δ*crzA*^CEA17^ strain than in the wild-type strain in the absence of caspofungin, but the contents were comparable in the two strains when exposed to caspofungin (Fig. 4B). In agreement, transmission electron microscopy (TEM) showed that in the absence of caspofungin, the cell wall of the Δ*crzA*^CEA17^ strain is about 3-fold thicker than that in the wild-type strain, whereas exposure to caspofungin did not result in any significant cell wall differences between the two strains (Fig. 5A and B). The increased cell wall thickness in the Δ*crzA*^CEA17^ strain may be the reason for the observed increased resistance to other cell wall-disturbing agents compared to the wild-type strain (Fig. S3A). These results indicate once more that CrzA also plays a role in cell wall composition in the absence of caspofungin and in the presence of other cell wall-stressing agents. This is in agreement with the ChIP-PCR results, where CrzA was also shown to bind to the promoter regions of *chsC*, *chsG*, and *csmB* (Fig. 3C).

10.1128/mBio.00705-17.3 FIG S3  (A) The Δ*crzA* strain is resistant to cell wall-damaging agents. Serial dilutions (10^5^ to 10^2^) of conidia of the wild-type (WT), Δ*crzA*^CEA17^, and Δ*crzA*::*crzA* complemented strains were grown on minimal medium supplemented with different concentrations of calcofluor white (CFW) or Congo red (CR) at 37°C for a few days. (B) Western blotting assays probing for MpkA phosphorylation in the CEA17 wild-type and Δ*crzA*^CEA17^ strains in the presence of increasing concentrations of caspofungin. Both strains were grown in complete medium for 18 h at 37°C before caspofungin was added for 60 min. Anti-p44/42 MpkA or anti-44/42 MpkA antibodies were used to detect the phosphorylation of MpkA (MpkA-P) and total MpkA, respectively. Anti-γ-tubulin antibody was used as a control for loading. A Coomassie brilliant blue (CBB)-stained gel is shown as an additional loading control. Signal intensities were quantified using the ImageJ software by dividing the intensity of MpkA-P/MpkA. Download FIG S3, PDF file, 0.3 MB. Copyright © 2017 Ries et al. 2017 Ries et al. This content is distributed under the terms of the Creative Commons Attribution 4.0 International license.

The phosphorylation of MpkA was not significantly altered in the Δ*crzA*^CEA17^ strain compared to the wild-type strain (Fig. S3B), suggesting that CrzA, in contrast to RlmA (13), is not involved in MpkA phosphorylation.

### The basic leucine zipper TF ZipD is involved in the CPE.

**FIG 6:**
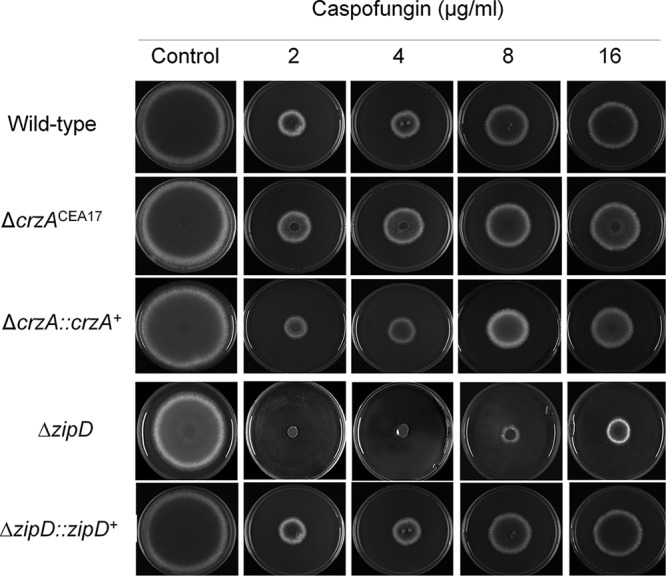
Deletion of *crzA* in strain CEA17 had no effect on the CPE response. *A. fumigatus* conidia (10^4^) were inoculated on solid minimal medium (MM) supplemented with glucose and different caspofungin concentrations and grown for 5 days at 37°C.

**FIG 7:**
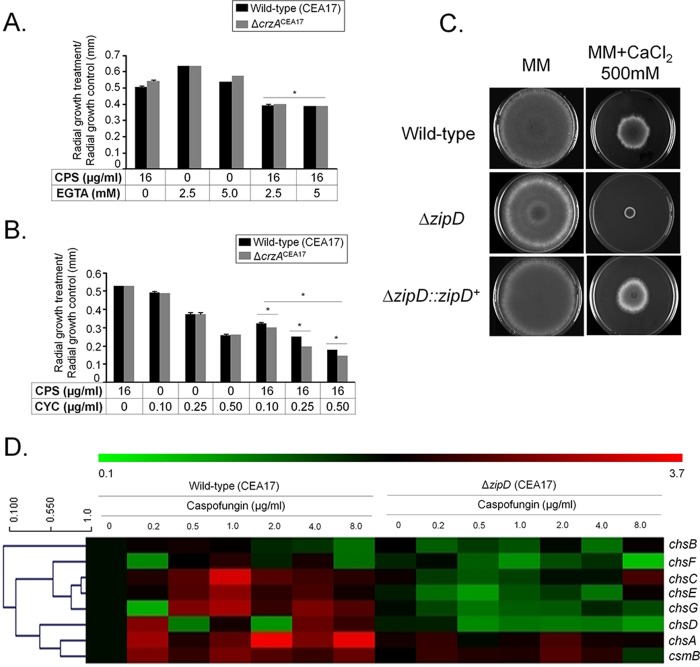
Identification of the transcription factor ZipD involved in the CPE. (A) The *A. fumigatus* wild-type (CEA17) and Δ*crzA*^CEA17^ strains were grown for 5 days at 37°C on solid minimal medium in the absence or presence of EGTA (2.5 and 5.0 mM) and caspofungin (CPS; 16 µg/ml). Results are expressed as the average of the radial diameter from three independent biological experiments of the treatment divided by the radial diameter of the control of three independent biological experiments (*, *P* < 0.001, as determined by *t* tests comparing the combined treatments to the single treatments). (B) Same as in panel A except that strains were grown in the presence of cyclosporine (CYC; 0.10, 0.25, and 0.50 µg/ml) and caspofungin (CPS; 16 µg/ml). (C) The wild-type CEA17, Δ*zipD*^CEA17^, and Δ*zipD*::*zipD*^+^ strains (10^4^ conidia) were grown for 5 days at 37°C on solid MM supplemented with 0 mM and 500 mM CaCl_2_. (D) Heat map and hierarchical linkage clustering (as determined by MeV software) of RT-qPCRs of the chitin synthase gene mRNA accumulation in the presence of caspofungin in the wild-type and Δ*zipD* strains. Strains were grown for 16 h at 37°C and transferred to increasing caspofungin concentrations for 60 min. The results are expressed as the average fold increase of the control cDNA (without caspofungin) for a specific gene from three independent biological experiments (with 2 technical repetitions each; see Fig. S1).

Surprisingly, in contrast to previous reports (19), where the deletion of *crzA* in the Af293 clinical isolate background strain abolished the CPE, deletion of the same gene in the CEA17 clinical isolate background strain had no effect on the CPE (Fig. 6). These results indicate strain-specific differences in the responses to cell wall-stressing agents. To determine whether the lack of the CPE in the Δ*crzA*^CEA17^ strain was still dependent on calcium signaling, the wild-type and Δ*crzA*^CEA17^ strains were grown in the presence of a calcium-chelating agent (e.g., EGTA) and the calcineurin-inhibiting agent cyclosporine. An increased inhibition in radial growth of the Δ*crzA*^CEA17^ strain, comparable to that in the wild-type strain, was observed when grown in the presence of subinhibitory concentrations of EGTA and high concentrations of caspofungin (Fig. 7A). Similarly, the combination of subinhibitory concentrations of cyclosporine and high concentrations of caspofungin resulted in a synergistic growth inhibition in both the wild-type and Δ*crzA*^CEA17^ strains (Fig. 7B). These results suggest that, although CrzA is not essential for the CPE, the cellular response to high concentrations of caspofungin is still dependent on calcium-mediated calcineurin signaling in the Δ*crzA*^CEA17^ strain.

**FIG 8:**
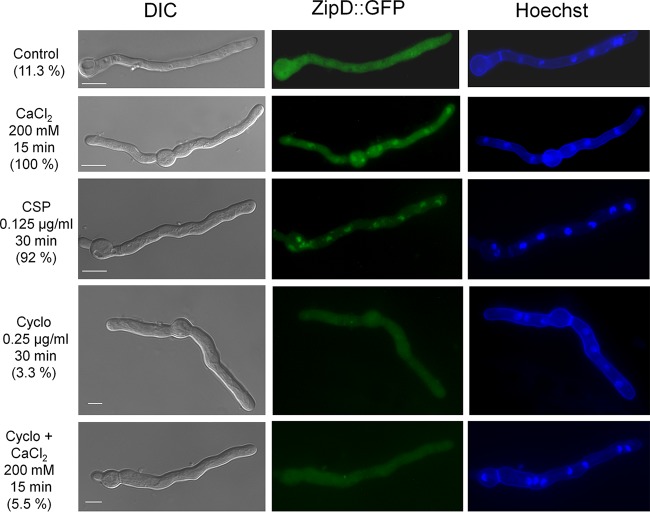
ZipD translocates to the nucleus in the presence of caspofungin and calcium. Cellular localization of the ZipD::GFP strain, as determined by microscopy, when grown for 16 h at 30°C and after incubation in the presence of CaCl_2_, caspofungin (CSP), cyclosporine (Cyclo), or both cyclosporine and CaCl_2_. The percentage of ZipD::GFP nuclear localization is indicated for each condition and based on counting between 300 and 600 nuclei of 50 to 100 hyphal germlings of biological triplicates. Bars, 5 μm.

A hypothesis for the maintenance of the CPE in the Δ*crzA*^CEA17^ strain but not in the Δ*crzA*^Af293^ strain may be the existence of additional calcium- and calcineurin-dependent TFs that mediate the regulation of the chitin synthase-encoding genes upon caspofungin exposure. Previously, four TFs encoded by the genes *htfA* (Afu4g10110), *zfpA* (Afu8g05010), *zfpB* (Afu1g10230), and *zipD* (Afu2g03280) were found to be upregulated in the presence of calcium (27, 28). Deletion of only *zipD* resulted in a reduced CPE and increased sensitivity to high levels of calcium (Fig. 6 and 7C). Moreover, the expression of chitin synthase-encoding genes was reduced in the Δ*zipD* (CEA17 background) strain compared to the wild-type strain upon exposure to increasing caspofungin concentrations (Fig. 7D and Fig. S1E). ZipD is a basic leucine zipper (bZIP, amino acids 85 to 154 as determined by SMART interface PF00170 [http://smart.embl-heidelberg.de/])-containing TF that is transcriptionally upregulated in the presence of calcium and negatively regulated by calcineurin (Fig. S4A). ZipD::GFP translocates to the nucleus in 100% and 92% of all counted hyphal germlings when they are incubated in high concentrations of calcium or caspofungin, respectively, a process which was shown to be calcineurin dependent (3.3% and 5.5%, respectively) (Fig. 8). Furthermore, the ZipD::GFP strain presented a growth phenotype similar to the wild-type strain in the absence and presence of high concentrations of calcium, indicating that ZipD is functional in this strain (Fig. S2A and B). Taken together, these results suggest that ZipD is involved in the calcium/calcineurin-dependent response to caspofungin by mediating directly or indirectly the transcriptional regulation of chitin synthase-encoding genes.

10.1128/mBio.00705-17.4 FIG S4  (A) The *zipD* gene is transcriptionally induced, as determined by RT-qPCR, by calcium and is dependent on calcineurin. The wild-type and Δ*calA* (calcineurin-deleted) strains were grown for 16 h at 37°C in minimal medium supplemented with glucose before being transferred to 200 mM CaCl_2_ for 10 and 30 min. The results are expressed as the fold increase of the expression relative to the control condition (no calcium). The *zipD* gene expression was normalized by β-tubulin gene (*βtubC*) expression. Standard deviations present the average from three independent biological repetitions each with 2 technical repetitions. Statistical analysis was performed using a one-way ANOVA with Dunnett’s *post hoc* test compared to the control condition (*, *P* < 0.05). (B) Amount of cell wall surface polysaccharides in the CEA17 and Af293 strains when grown for 16 h in minimal medium supplemented with glucose (dectin recognizes β-1,3-glucan, CFW recognizes chitin, SBA-FITC recognizes galactosaminogalactan [GAG], WGA-FITC recognizes glucosamine, and ConA-FITC recognizes mannose). Download FIG S4, PDF file, 0.1 MB. Copyright © 2017 Ries et al. 2017 Ries et al. This content is distributed under the terms of the Creative Commons Attribution 4.0 International license.

## DISCUSSION

The *A. fumigatus* CPE has been observed for more than a decade, with calcium signaling (19, 20) and the chaperone Hsp90 (21, 22) shown to play an important role in the subsistence of the CPE, although the exact mechanistic nature underlying this phenomenon remains largely uncharacterized. The comprehension of the molecular events governing the CPE provides an opportunity to uncover new factors involved in the biosynthesis of cell wall components, which are important during infection due to their immunomodulatory and immunoevasive properties (9). This study therefore set out to further investigate the molecular mechanisms underlying the *in vitro* CPE in an *A. fumigatus* clinical isolate.

Exposure to lower CPE concentrations activated the CWI pathway, as shown by increased phosphorylation of the CWI MAPK MpkA, whereas much higher CPE concentrations did not result in MpkA phosphorylation, suggesting an inactivation of the CWI pathway in liquid medium in these concentrations. In agreement, deletion of *mpkA* and its downstream transcriptional regulator *rlmA* resulted in the loss of the CPE on solid medium, although at higher caspofungin concentrations than those used during the phosphorylation assay. The onset of the CPE is significantly different between solid and liquid assays, with the hyphae presenting increased sensitivity to caspofungin in liquid cultures, which may explain the above-observed discrepancy. Furthermore, it cannot be excluded that additional MpkA phosphorylation patterns may be observed in liquid cultures at shorter or longer incubation times at higher caspofungin concentrations which would transiently induce the CWI pathway.

The absence of the CPE in the Δ*mpkA* strain does not result from a lack of transcriptional upregulation of chitin synthase-encoding genes, a mechanism which is thought to mediate protection/resistance to caspofungin (14–17). Actually, MpkA rather seems to exert a repressive function on some chitin synthase-encoding genes at higher caspofungin concentrations, confirming that MpkA plays a role in the regulation of these genes. It is possible that MpkA participates in the (positive) regulation of other cell wall biosynthetic genes, as was shown in reference 13, where MpkA is involved in regulating the expression of β-1,3- and α-1,3-glucan synthases and 1,3-β-glucanosyltransferases. In agreement, it was recently proposed that initial growth of *A. fumigatus* in the presence of high concentrations of caspofungin requires chitin synthesis but that the paradoxical growth effect relies on the reactivation of the β-1,3-glucan synthase during the later stages of growth in the presence of caspofungin (18). The role of MpkA in regulating cell wall biosynthetic genes during CPE and in the presence of different cell wall stresses, as well as downstream targets, remains to be determined in future studies. To date, only RlmA has been identified as a direct target of MpkA in the presence of Congo red, a dye which prevents correct glucan formation (13), although RlmA does not appear to be targeted by MpkA in the presence of caspofungin. In contrast to MpkA, RlmA is involved in the positive regulation of some chitin synthase-encoding genes in the presence of higher caspofungin concentrations, suggesting an MpkA-independent function of RlmA. Furthermore, these results also suggest specific responses to different cell wall-perturbing agents, likely mediated by additional transcription factors that together regulate the appropriate response to various cell wall perturbations.

In *A. fumigatus*, as in *C. albicans*, the upregulation of chitin synthase-encoding genes during the CPE is dependent on calcineurin signaling (19, 29). Subsequent chitin synthase promoter analysis and *in vitro* and *in vivo* binding studies identified the calcium/calcineurin-dependent TF CrzA to play a role in the CPE by directly binding to and regulating the expression of certain chitin synthase-encoding genes. These results are in agreement with reference 19, which predicted regulation of two chitin synthase-encoding genes by CrzA, although direct evidence for gene promoter binding was not provided. In a previous study, ChIP-seq (chromatin immunoprecipitation coupled to DNA sequencing) of CrzA did not identify any cell wall biosynthesis gene targets, which is probably due to the different growth conditions (calcium- versus caspofungin-treated mycelia) used (26). In agreement with the promoter binding studies, CrzA translocation to the nucleus is calcineurin dependent in the presence of different concentrations of caspofungin. Taken together, these results suggest that chitin synthase gene regulation is governed by multiple parallel-acting and/or cross-talking (e.g., CWI and calcium/calcineurin) cellular signaling pathways. In *C. albicans*, chitin biosynthesis is regulated via protein kinase C (PKC) signaling, the high-osmolarity glycerol (HOG) pathway, and calcium/calcineurin signaling (19). A similar situation can be envisaged for *A. fumigatus*, where CrzA has also been shown to be involved in the regulation of HOG pathway intermediates (26), therefore providing a link between calcium signaling and the HOG pathways. Furthermore, *in silico* analysis of mycelia grown in subinhibitory caspofungin concentrations predicted a cross talk between the HOG MAPK SakA and the CWI MAPK MpkA (30).

In contrast to the *A. fumigatus* clinical isolate Af293 (19), deletion of *crzA* in the clinical isolate CEA17 did not result in the loss of the CPE, indicating strain-specific differences in the response to high concentrations of caspofungin. In *C. albicans*, growth on echinocandins was shown to be strain specific (31), and strain-specific responses of different *A. fumigatus* clinical isolates to various caspofungin concentrations have also been observed (13). In agreement, deletion of *crzA* in the Af293 background strain resulted in a much more severe growth phenotype under nonstress conditions than in the CEA10 background strain (27, 32). Furthermore, differences in virulence in a triamcinolone murine model of invasive pulmonary aspergillosis (IPA) as well as differences in growth phenotypes under normoxic and hypoxic conditions have also been observed between strains CEA10 and Af293 (33), further supporting the notion that fundamental differences exist between these two strains. These differences may be related to the composition of cell wall surface polysaccharides which showed significant differences in the organization and exposure of galactosaminogalactan (GAG), chitin, β-1,3-glucan, glucosamine, and mannose between the two strains (Fig. S4B). The exact genetic and molecular basis underlying these strain-specific differences was beyond the scope of this work and is the subject of further research. In addition, the growth phenotype of the Δ*crzA* strain on minimal medium supplemented with glucose differed in this study from that in reference 31, which is likely due to different incubation times (5 days versus 2 days) and amounts of conidia used during initial inoculation (10^5^ versus 10^3^ conidia). The CPE in strain CEA17 was determined to still be calcium signaling dependent, suggesting that other factors are involved in the maintenance of the CPE. Recently, SakA^Hog1^-dependent TFs, such as the *ATF1* homologues AtfA, -B, and -D, were shown to be involved in the CPE in *A. fumigatus* (34). In agreement, an additional TF, termed ZipD, was identified as functioning in the calcium-calcineurin pathway in strain CEA17 and as being involved in mediating the CWI response to caspofungin by regulating (directly or indirectly) cell wall enzyme-encoding genes. Future work will focus on identifying potential ZipD binding sites and targets.

In summary, this work highlights the importance of calcium metabolism in the CPE and begins to unravel the immense complexity underlying the regulation of cell wall biosynthesis genes when the fungus is exposed to cell wall-stressing agents, such as caspofungin. Several cellular signaling pathways are engaged upon *A. fumigatus* exposure to caspofungin, resulting in the activation of different TFs which regulate cell wall biosynthesis. Although the CPE has mainly been studied *in vitro*, there is evidence for clinical relevance, as determined by increased pulmonary fungal burden in a murine model of aspergillosis when administered high doses of caspofungin (35). Additional characterization of the target genes regulated by MpkA, RlmA, CrzA, and ZipD as well as reconstruction of the molecular mechanisms involved in the cell wall compensatory pathways during the CPE could ultimately provide an opportunity for the development of new antifungal therapies. Combinatory antifungal therapies are considered an emerging strategy in successfully treating IA (36). Considering that calcineurin inhibitors are active *in vitro* against *A. fumigatus* and act synergistically with caspofungin (37), combinations of fungal calcineurin-specific and/or chitin synthase inhibitors together with minimal concentrations of echinocandins could provide a therapy for effectively combating IA.

## MATERIALS AND METHODS

### Strains and media.

All strains used in this study are listed in Table S2 in the supplemental material. Strains were grown at 37°C in either complete medium (CM; 2% [wt/vol] glucose, 0.5% [wt/vol] yeast extract, trace elements) or minimal medium (MM; 1% [wt/vol] glucose, original high-nitrate salts, trace elements, pH 6.5). Solid CM and MM were the same as described above except that 1.7% (wt/vol) or 2% (wt/vol) agar was added. Where necessary, uridine and uracil (1.2 g/liter) were added. Trace elements, vitamins, and nitrate salt compositions were as described previously (38).

10.1128/mBio.00705-17.6 TABLE S2  Strains used in this study. Download TABLE S2, PDF file, 0.6 MB. Copyright © 2017 Ries et al. 2017 Ries et al. This content is distributed under the terms of the Creative Commons Attribution 4.0 International license.

### Microscopy.

Strains were left to germinate on coverslips in 4 ml of CM for 16 h at 30°C before caspofungin was added (Fig. 1 and 4 give concentrations and incubation times). Coverslips were washed with phosphate-buffered saline (PBS; 140 mM NaCl, 2 mM KCl, 10 mM NaHPO_4_, 1.8 mM KH_2_PO_4_, pH 7.4) and stained for 3 min in 12 μg/ml Hoechst stain (Life Technologies, Inc.). Coverslips were washed again with PBS and visualized with an observer Z1 fluorescence microscope using the 100× objective oil immersion lens (for GFP, filter set 38 [high efficiency], excitation wavelength of 450 nm to 490 nm, and emission wavelength of 525 nm to 550 nm; for Hoechst/DAPI [4′,6-diamidino-2-phenylindole] stain, filter set 49, excitation wavelength of 365 nm, and emission wavelength of 420 nm to 470 nm). Differential interference contrast (DIC) images and fluorescent images were captured with an AxioCam camera (Carl Zeiss, Inc.) and processed using the AxioVision software (version 4.8).

### Immunoblot analysis.

Strains were grown from 1 × 10^7^ conidia at 37°C, 200 rpm, in CM for 16 h before being exposed to increasing concentrations of caspofungin for 60 min. Proteins were extracted as described previously (19) and quantified according to the Lowry method modified by Hartree (39). Sixty micrograms of total protein per sample was run on a 10% (wt/vol) SDS-PAGE gel before being transferred to a polyvinylidene difluoride (PVDF) membrane (Bio-Rad). Phosphorylated MpkA was detected using anti-phospho-p44/42 MAPK antibody (9101; Cell Signaling Technologies), whereas the total amount of MpkA was detected using the anti-p44/42 MAPK antibody (4370; Cell Signaling Technologies) according to the manufacturer’s instructions. Secondary antibody was peroxidase conjugated (A0545 [Sigma] or sc-2020 [Santa Cruz Biotechnology], respectively), and anti-γ-tubulin (yN-20; Santa Cruz Biotechnology) was used as the loading control. Detection (chemiluminescence) was performed using the ECL Prime Western blot detection kit (GE Healthcare), according to the manufacturer’s instructions. Images were generated by exposing the PVDF membranes to the ChemiDoc XRS gel imaging system (Bio-Rad) and subjected to densitometric analysis in ImageJ software (40) to calculate the phosphorylated MAPK/total MAPK ratio.

### TEM analysis of cell wall.

Strains were grown statically from 1 × 10^7^ conidia at 37°C in MM for 24 h before being exposed to 0.125 µg/ml of caspofungin for 120 min. Mycelia were harvested and immediately fixed in 0.1 M sodium phosphate buffer (pH 7.4) containing 2.5% (vol/vol) glutaraldehyde and 2% (wt/vol) paraformaldehyde for 24 h at 4°C. Samples were encapsulated in agar (2% [wt/vol]) and subjected to fixation (1% OsO_4_), contrasting (1% uranyl acetate), ethanol dehydration, and a two-step infiltration process with Spurr resin (Electron Microscopy Sciences) of 16 h and 3 h at room temperature (RT). Additional infiltration was provided under vacuum at RT before embedment in Beem capsules (Electron Microscopy Sciences) and polymerization at 60°C for 72 h. Semithin (0.5-µm) survey sections were stained with toluidine blue to identify the areas of best cell density. Ultrathin sections (60 nm) were prepared and stained again with uranyl acetate (1%) and lead citrate (2%). Transmission electron microscopy (TEM) images were obtained using a Philips CM-120 electron microscope at an acceleration voltage of 120 kV using a MegaView3 camera and iTEM 5.0 software (Olympus Soft Imaging Solutions GmbH). Cell wall thicknesses of 200 sections of different germlings were measured at ×23,500 magnification, and images were analyzed with the ImageJ software (40). Statistical differences were evaluated by using one-way analysis of variance (ANOVA) and Tukey’s *post hoc* test.

### Extraction and sugar quantification of cell wall polysaccharides.

Fungal cell wall polysaccharides were extracted from 10 mg dry-frozen biomass as described previously (41). One milliliter of extracted samples was concentrated 10× by lyophilization, and sugars were subsequently analyzed by high-performance liquid chromatography (HPLC) using a YoungLin YL9100 series system (YoungLin, Anyang, South Korea) equipped with a YL9170 series refractive index (RI) detector at 40°C. Samples were loaded in the Rezex ROA (Phenomenex, USA) column (300 by 7.8 mm) at 85°C and eluted with 0.05 M sulfuric acid at a flow rate of 1.5 ml/min.

### RNA extraction and gene expression analysis.

Strains were grown from 1 × 10^7^ conidia in CM for 16 h at 37°C before being incubated with increasing concentrations of caspofungin (0.2 µg/ml to 8 µg/ml) for 60 min. Mycelia were ground to a fine powder in liquid N_2_, and total RNA was extracted with Trizol reagent (Thermo Scientific) according to the manufacturer’s protocol. DNA was digested with Turbo DNase I (Ambion Thermo Scientific) according to the manufacturer’s instructions. Two micrograms of total RNA per sample was reverse transcribed with the high-capacity cDNA reverse transcription kit (Thermo Scientific) using oligonucleotide dT (deoxythymine) and a random blend of primers, according to the manufacturer’s instructions. RT-qPCRs were run in a StepOne Plus real-time PCR system (Thermo Scientific), and Power Sybr green PCR master mix (Thermo Scientific) was used. Three independent biological replicates were used, and the mRNA quantity relative fold change was calculated using the threshold cycle (2^−ΔΔ*CT*^) method (42). All values were normalized by the expression of the *A. fumigatus* β-tubulin gene. Primers used for the *A. fumigatus* chitin synthase genes (*chsA* to -*G* and *csmB*) are described in reference 43.

### ChIP-qPCR.

The wild-type CEA17 and CrzA::GFP strains were grown for 24 h in MM supplemented with 1% (wt/vol) glucose before caspofungin was added to a final concentration of 0, 0.2, 2, and 8 μg/ml for 1 h. Cross-linking and sonication of the samples were carried out as described previously (44). Briefly, samples were cross-linked with 1% (vol/vol) formaldehyde at 30°C for 20 min before formaldehyde was quenched with 2 M glycine for 10 min at 30°C. Mycelia were snap-frozen, ground to a fine powder in liquid N_2_, and resuspended in 3 to 4 ml ChIP lysis buffer (45). Two milliliters of samples was sonicated as described previously (44), centrifuged for 5 min at 4°C, and stored at −80°C. Sonication was checked in 60 μl of reverse cross-linked material for each sample.

Immunoprecipitation (IP) was then carried out using 900 μl of the sonicated sample and 20 μl of the GFP Trap_A resin (ChromoTek, Planegg-Martinsried, Germany). Resin equilibration, incubation, and washes were carried out as described previously (45). Proteins were released from the resin by incubating it two times with 50 μl elution buffer at 65°C before samples were reverse cross-linked overnight at 65°C as described previously (44). DNA was purified using the QIAquick gel extraction and PCR purification kit (Qiagen) according to the manufacturer’s instructions.

The qPCRs were carried out as described previously (45). One microliter of sonicated and purified DNA was used per 20-μl reaction mixture. Primers used to amplify *chsA*, *chsC*, *chsG*, and *csmB* are shown in Table S3. Cross-linked but nonsonicated samples (input) were used as positive controls, and gene expression was calculated and normalized according to the percent input method (Thermo Fisher).

10.1128/mBio.00705-17.7 TABLE S3  Oligonucleotides used in this work (ORF, open reading frame). Download TABLE S3, PDF file, 0.1 MB. Copyright © 2017 Ries et al. 2017 Ries et al. This content is distributed under the terms of the Creative Commons Attribution 4.0 International license.

### EMSAs, DNA probes, and competitors.

EMSAs were carried out using a purified GST-tagged CrzA protein according to the procedure described in reference 25. DNA fragments corresponding to the promoter regions containing the CrzA *cis-*regulatory element of the target genes were prepared (described below) and assayed in 30 μl of 1× EMSA binding buffer, also containing 3 µg of the nonspecific competitor poly(dI⋅dC) (GE Healthcare) and 3 and 6 µg of the GST::CrzA recombinant protein. The reaction mixtures were incubated with the radiolabeled probes (~10^4^ cpm) at RT for 30 min being run on a 5% polyacrylamide gel in 0.5× Tris-borate EDTA (TBE) buffer at 300 V at 10°C. The DNA-protein complexes were detected by autoradiography after exposing the dried gels to X-ray films. For competition assays, a 10-fold molar excess of the nonlabeled DNA fragments (specific competitor) was added to the binding reaction mixtures 10 min prior to the incubation with the respective radiolabeled probes.

Probes and specific competitors of the *chsA*, *csmB*, *chsC*, and *chsG* promoters, containing the *cis*-regulatory CrzA consensus (26), were amplified by PCR in the presence and absence of [α-^32^P]dATP (3,000 Ci/mmol). The *chsA*, *csmB*, *chsC*, and *chsG* probes were amplified using the following primer pairs: *chsA*-F/*chsA*-R (144 bp), *csmB*-F/*csmB*-R (155 bp), *chsC*-F/*chsC*-R (155 bp), and *chsG*-F/*chsG*-R (167 bp) (Table S3). The amplified DNA fragments were purified from 2% (wt/vol) low-melting-point agarose gels and quantified at 260 nm, and the counts per minute was counted by liquid scintillation. To confirm the specificity of the DNA-protein interactions, mutated probes were constructed by site-directed mutagenesis in a two-step PCR. The m*chsA*, m*csmB*, m*chsC*, and m*chsG* mutated probes were constructed using the following primer pairs in the first PCR: m*chsA*-F/m*chsA*-R, m*csmB*-F/m*csmB*-R, m*chsC*-F/m*chsC*-R, and m*chsG*-F/m*chsG*-R (Table S3). The same primer pairs as described above were used for the second-step PCR.

### Staining for dectin-1, chitin, and other cell surface carbohydrates.

Cell wall surface polysaccharide straining was performed as described previously (46, 47). Briefly, strains were grown from 2.5 × 10^3^ spores in 200 µl of MM for 16 h at 37°C before the culture medium was removed and germlings were UV irradiated (600,000 µJ). Hyphal germlings were subsequently washed with PBS before 200 µl of a blocking solution (2% [wt/vol] goat serum, 1% [wt/vol] bovine serum albumin [BSA], 0.1% [vol/vol] Triton X-100, 0.05% [vol/vol] Tween 20, 0.05% [vol/vol] sodium azide, and 0.01 M PBS) was added, and samples were incubated for 30 min at room temperature (RT).

For dectin staining, 0.2 µg/ml of Fc-h-dectin-hFc was added to the UV-irradiated germlings and incubated for 1 h at RT, followed by the addition of 1:1,000 DyLight 594-conjugated, goat anti-human IgG1 for 1 h at RT (46). Germlings were washed with PBS, and fluorescence was read at 587-nm excitation and 615-nm emission. For chitin staining, 200 µl of a PBS solution with 10 µg/ml of calcofluor white (CFW) was added to the UV-irradiated germlings, incubated for 5 min at RT, and washed three times with PBS before fluorescence was read at 380-nm excitation and 450-nm emission. For galactosaminogalactan (GAG), GlcN (glucosamine), and mannose staining, 200 µl of PBS supplemented with 0.1 mg/ml of either soybean agglutinin-fluorescein isothiocyanate (SBA-FITC) (*Glycine max* soybean lectin SBA-FITC; Bioworld; catalog no. 21761024-2), wheat germ agglutinin (WGA) (lectin-FITC L4895; Sigma), or concanavalin A (ConA; C7642; Sigma) was added to the UV-irradiated germlings for 1 h at RT. Germlings were washed with PBS, and fluorescence was read at 492-nm excitation and 517-nm emission. All experiments were performed using 12 repetitions, and fluorescence was read in a microtiter plate reader (SpectraMax i3; Molecular Devices).
