# Supplementary figures and images for: The Aspergillus fumigatus CrzA Transcription Factor Activates Chitin Synthase Gene Expression during the Caspofungin Paradoxical Effect
Source: mBio. 2017 Jun 13;8(3):e00705-17. doi: 10.1128/mBio.00705-17 (PMC5472186; doi:10.1128/mBio.00705-17)

A.

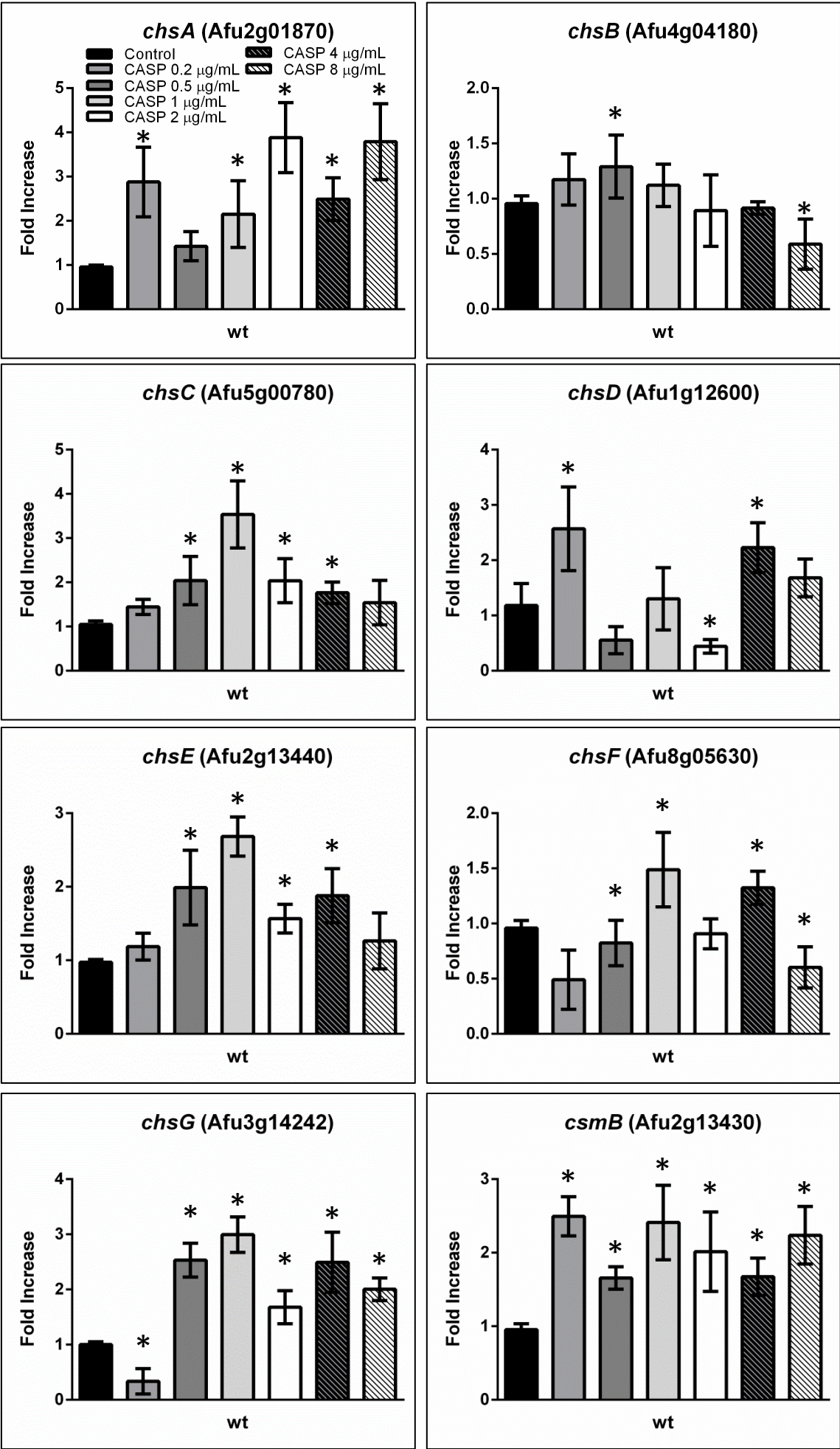

B.

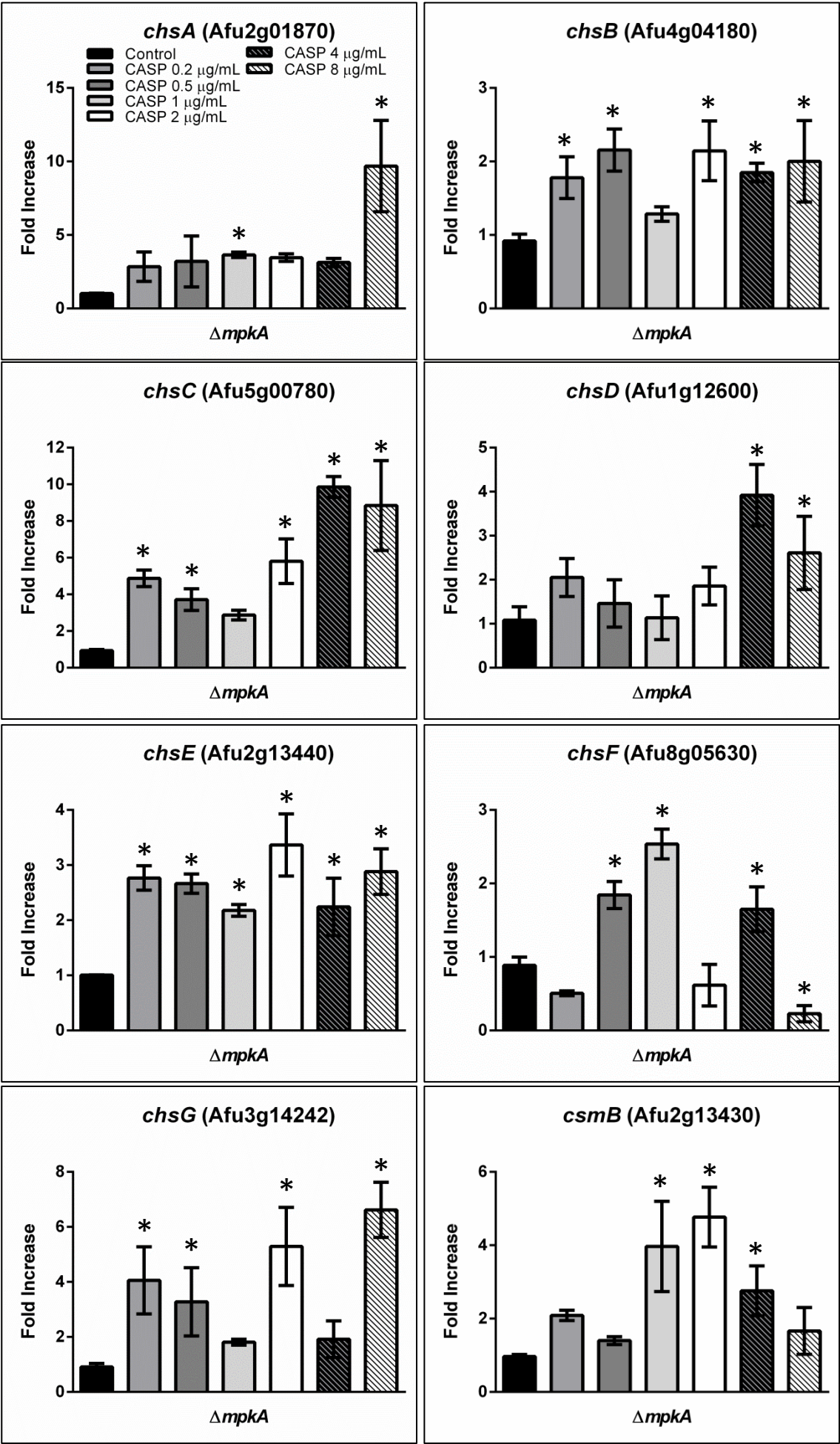

C.

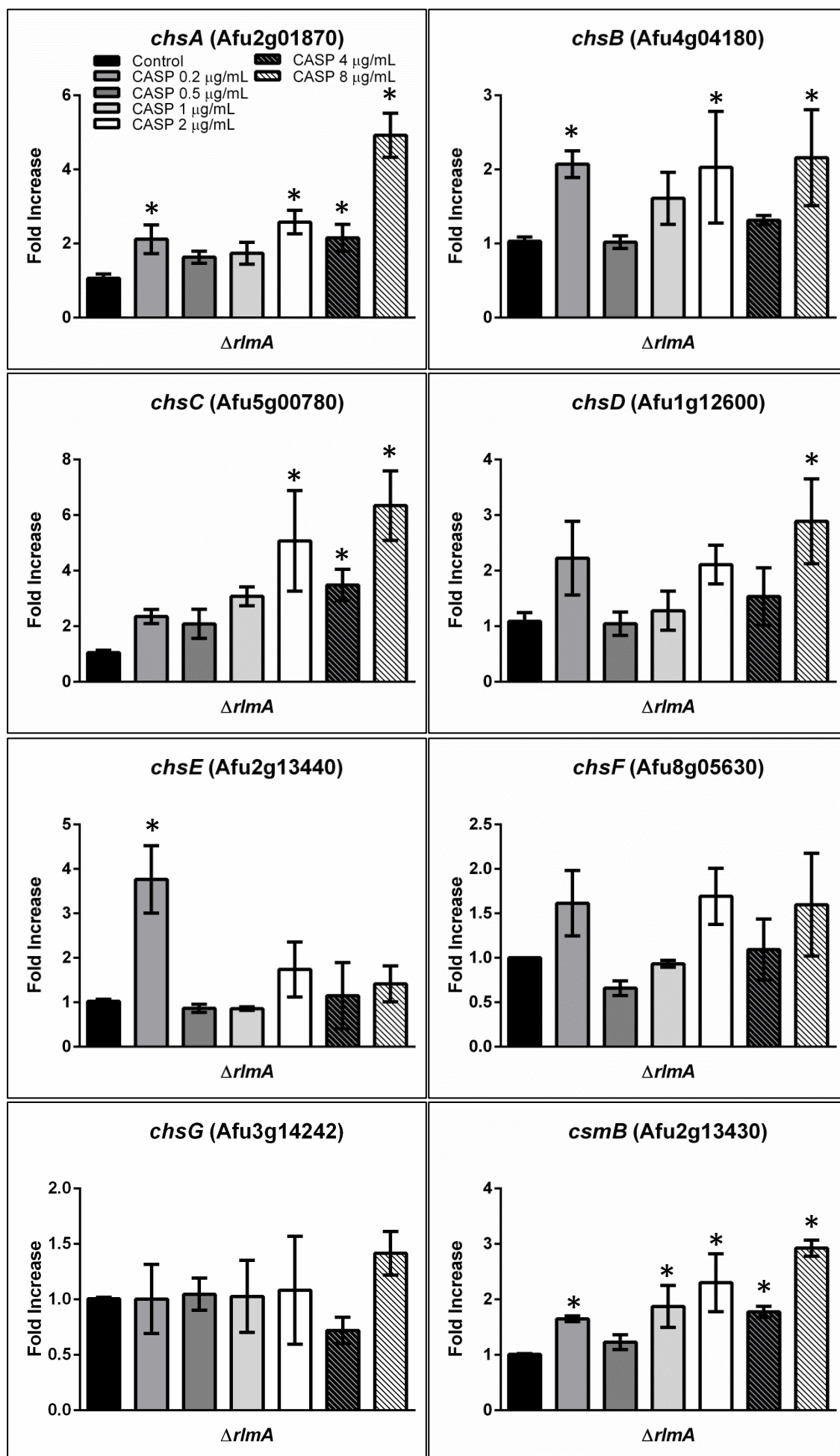

D.

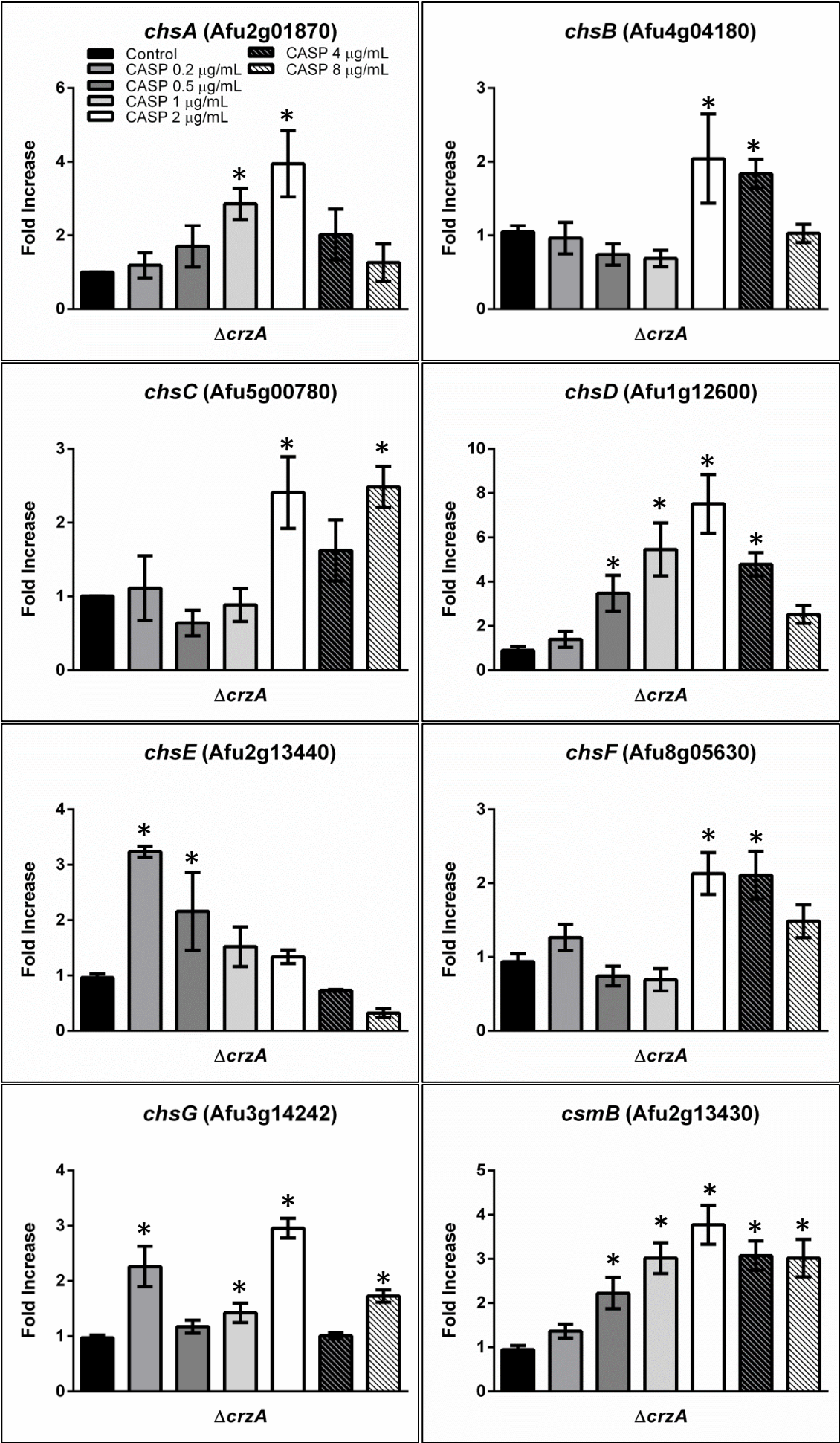

E.

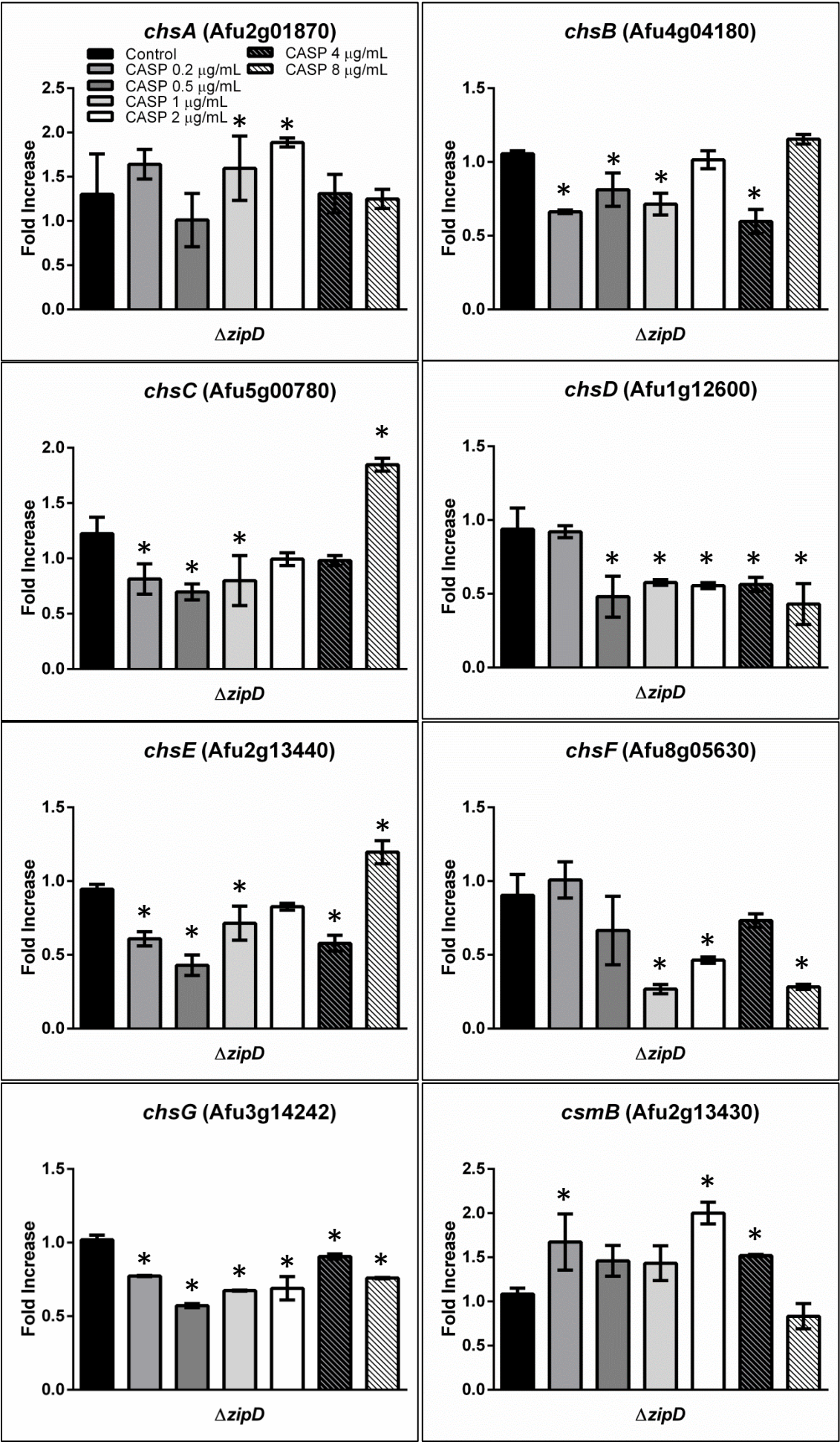

Supplement: FIG S1 [file mbo003173337sf1.pdf]

A.

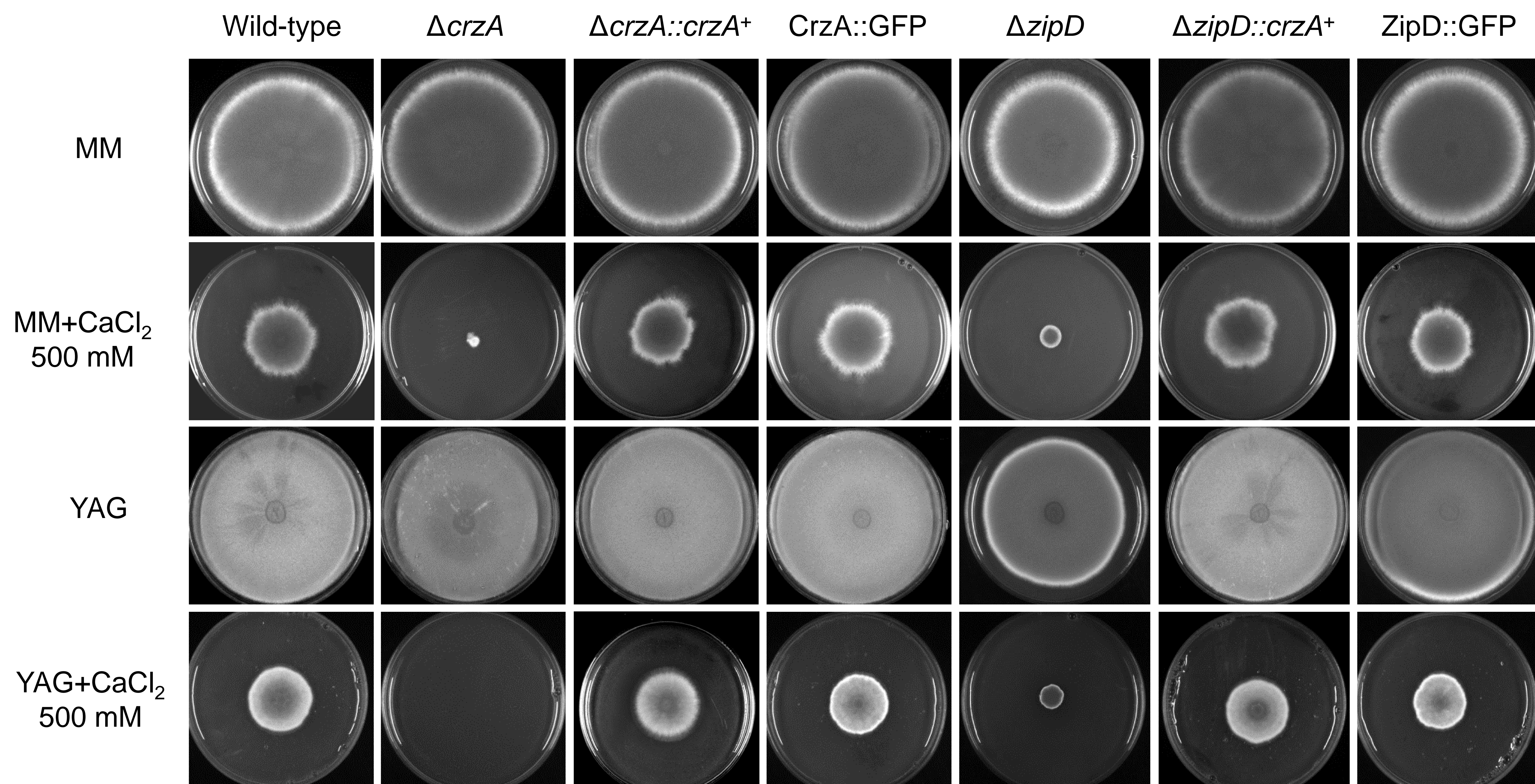

B.

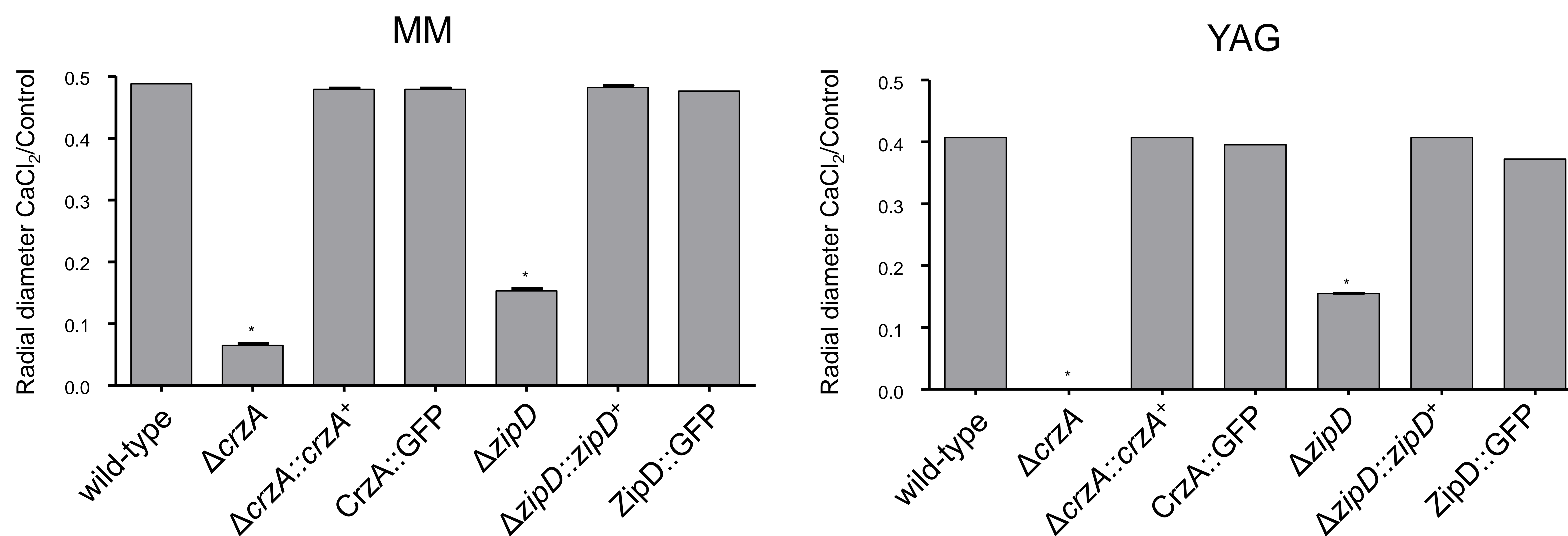

C.

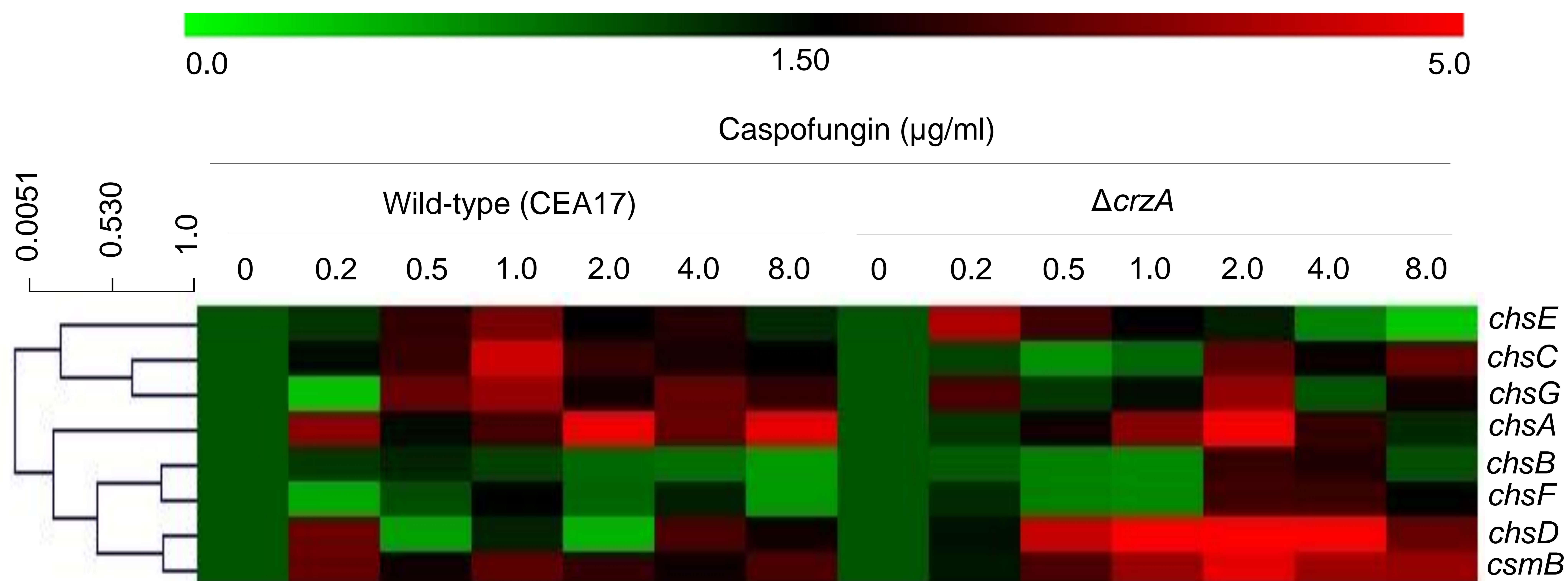

Supplement: FIG S2 [file mbo003173337sf2.pdf]

A.

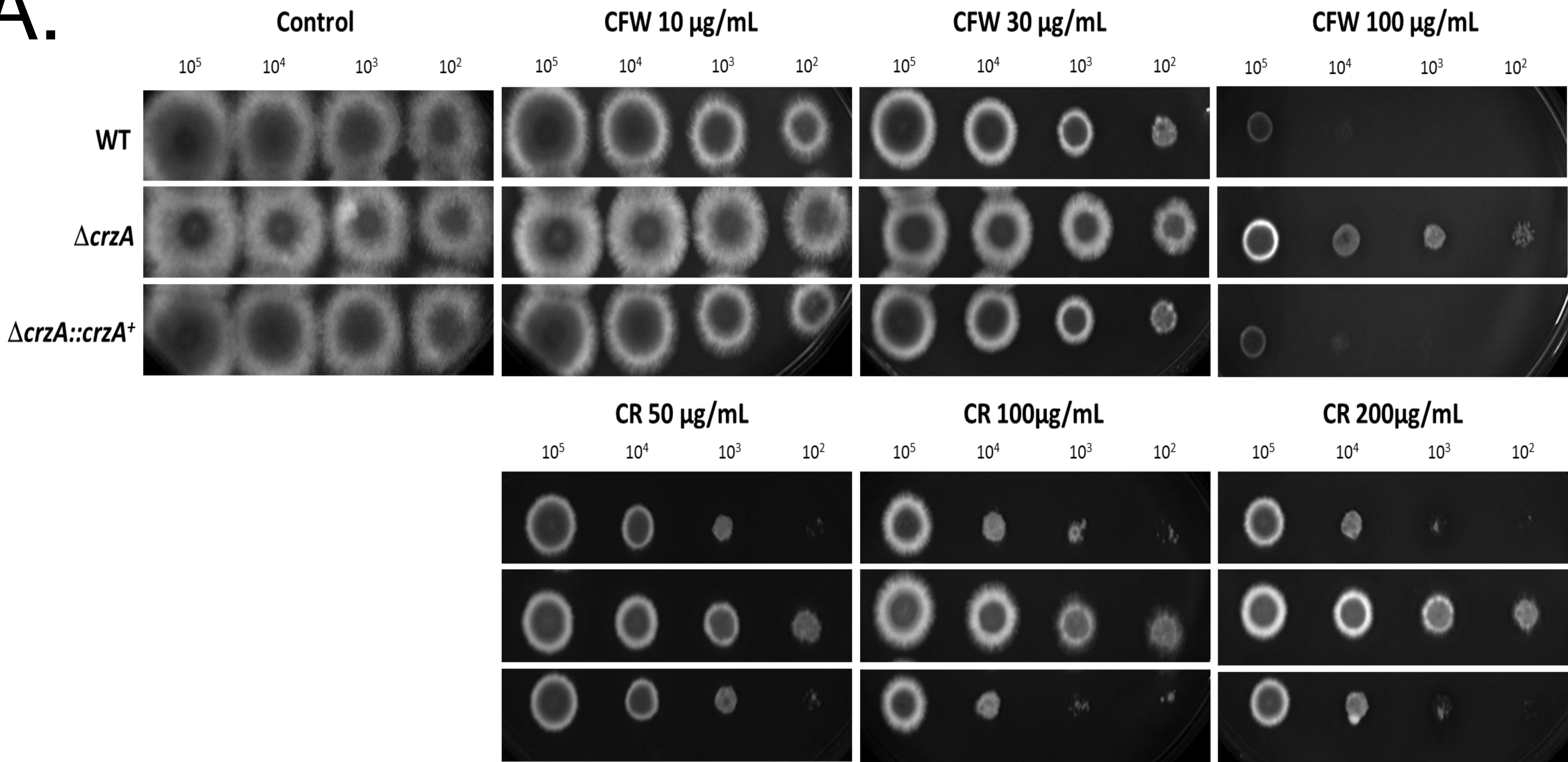

B.

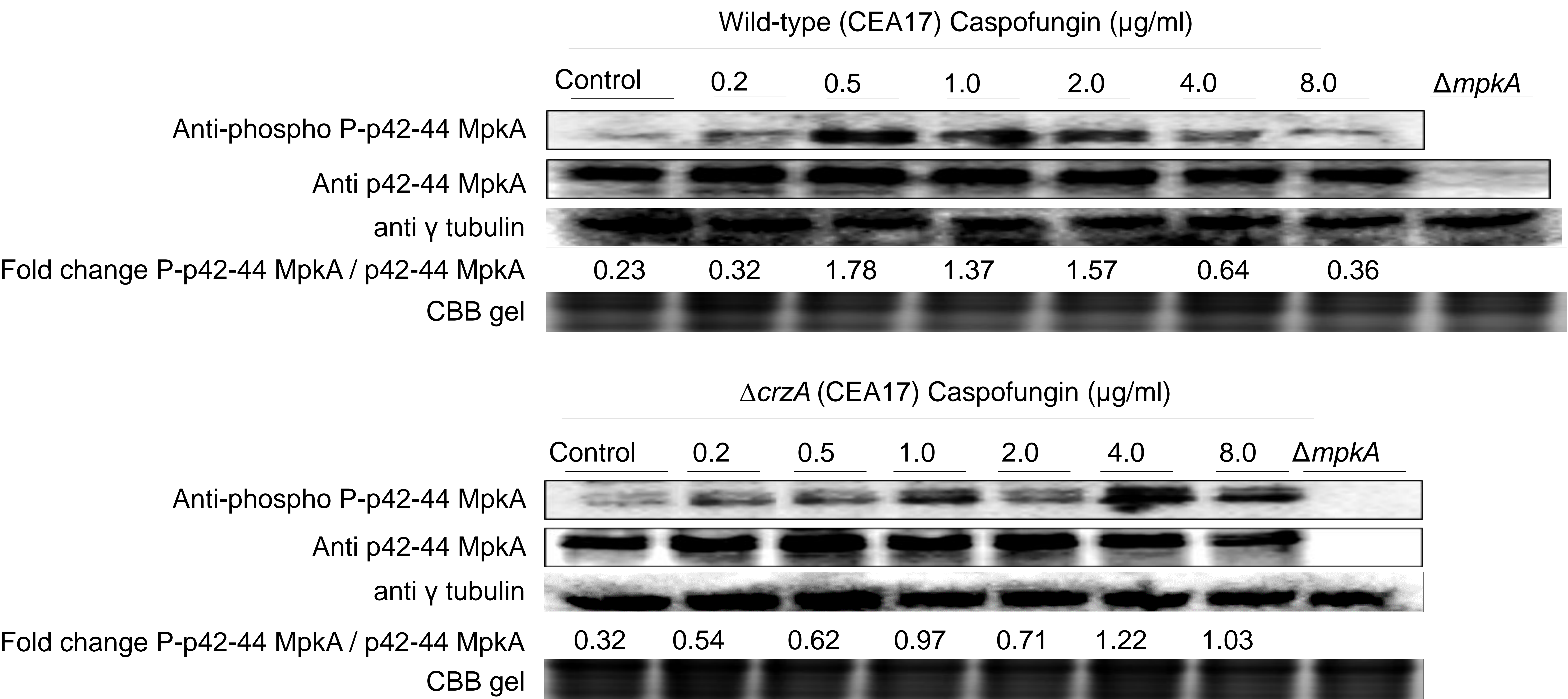

Supplement: FIG S3 [file mbo003173337sf3.pdf]

A.

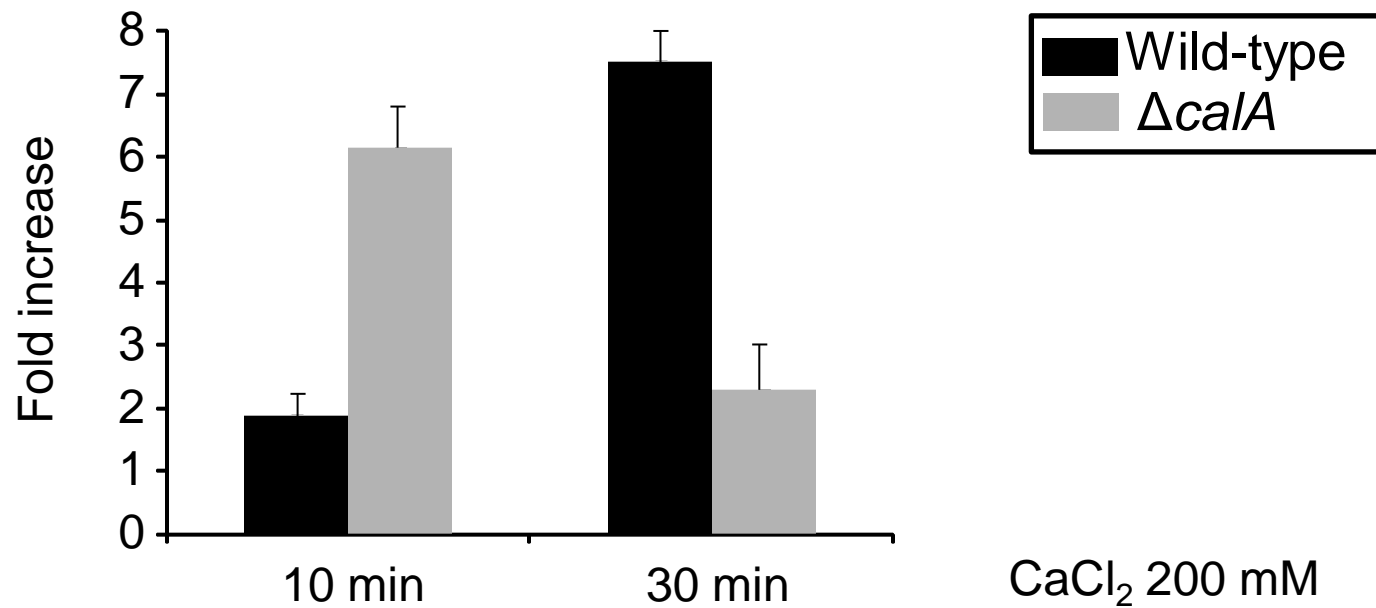

B.

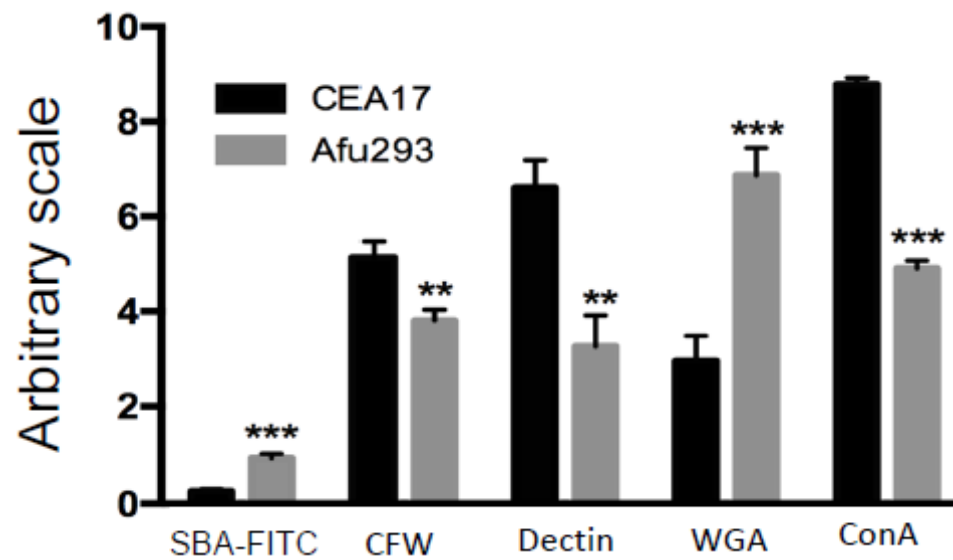

Supplement: FIG S4 [file mbo003173337sf4.pdf]
