# Supplementary material for: The Aspergillus fumigatus CrzA Transcription Factor Activates Chitin Synthase Gene Expression during the Caspofungin Paradoxical Effect
Source: mBio. 2017 Jun 13;8(3):e00705-17. doi: 10.1128/mBio.00705-17 (PMC5472186; doi:10.1128/mBio.00705-17)
Supplement: TABLE S1 [file mbo003173337st1.pdf]

Consensus sequence and in which promoter region identified	Gene strand	Start	P-value	Motif for known transcription factor
**1.** CTCAGC[CT][AC]TC				
chsA	-	184	1.52e-06	
chsB	-	172	3.98e-06	
chsC	+	636	8.13e-07	
chsE	+	739	2.45e-06	
chsF	+	638	1.52e-06	
chsG	-	467	8.09e-06	
csmB	+	944	1.17e-05	UNKNOWN
**2.** CTTCC[AC]T[CA]CT				
csmB	+	454	9.32e-07	
chsD	+	520	2.81e-06	
chsF	-	122	6.30e-06	
chsE	+	536	9.37e-06	
chsC	-	505	9.37e-06	
chsA	+	818	1.02e-05	
chsB	+	343	1.16e-05	
chsG	+	706	1.27e-05	*SFL1* (MA0377.1)<br>*GCR2* (MA0305.1)<br>*HSF1* (MA0319.1)<br>*GCR1* (MA0304.1)

**Consensus sequence and in which promoter region identified**	**Gene strand**	**Start**	**P-value**	**Motif for known transcription factor**
**3.** TCA[GT]CCAC				
chsF	+	563	1.31e-05	
chsD	+	184	1.31e-05	
chsC	+	356	1.31e-05	
chsG	+	694	2.80e-05	
chsA	+	626	2.80e-05	
chsE	-	832	4.11e-05	
csmB	+	763	5.42e-05	
chsB	-	884	6.73e-05	*CRZ1* (MA0285.1)
**4.** T[TC]T[TC]CCCTTG				
chsF	+	287	1.07e-06	
csmB	-	816	2.93e-06	
chsG	+	898	2.93e-06	
chsD	-	267	5.86e-06	
chsB	-	313	1.00e-05	
chsE	+	669	1.89e-05	
chsA	+	248	1.89e-05	
chsC	+	35	2.37e-05	UNKNOWN
**5.** TTTTT[CT]TTT				
chsG	+	746	6.02e-06	
chsF	+	345	6.02e-06	
chsB	-	528	6.02e-06	
csmB	+	547	1.29e-05	
chsE	+	472	1.29e-05	
chsC	+	14	1.29e-05	
chsD	-	119	2.49e-05	
chsA	-	121	2.49e-05	*AZF1* (MA0277.1)<br>*SFP1* (MA0378.1)

Consensus sequence and in which promoter region identified	Gene strand	Start	P-value	Motif for known transcription factor
**6.** GC[CG]G[AT]CACTC				
chsG	+	34	1.42e-06	
chsF	+	274	1.42e-06	
chsB	-	935	1.42e-06	
chsC	-	188	2.84e-06	UNKNOWN
**7.** T[GC]TTGAT[CG]GA				
chsG	+	815	1.07e-06	
chsF	+	942	3.20e-06	
chsB	-	792	3.20e-06	
csmB	-	805	6.58e-06	
chsD	+	848	9.79e-06	
chsC	-	699	1.53e-05	
chsE	-	345	2.20e-05	
chsA	-	968	3.33e-05	UNKNOWN
**8.** T[TC][TG][CG]CA[CG][TC]G[TG]				
chsG	+	936	2.14e-06	
chsE	+	777	5.86e-06	
chsA	+	287	5.86e-06	
csmB	+	592	6.93e-06	
chsF	+	251	1.07e-05	
chsC	+	165	1.07e-05	
chsD	-	889	1.66e-05	
chsB	-	763	3.67e-05	UNKNOWN
