# Supplementary material for: The Aspergillus fumigatus CrzA Transcription Factor Activates Chitin Synthase Gene Expression during the Caspofungin Paradoxical Effect
Source: mBio. 2017 Jun 13;8(3):e00705-17. doi: 10.1128/mBio.00705-17 (PMC5472186; doi:10.1128/mBio.00705-17)
Supplement: TABLE S2 [file mbo003173337st2.pdf]

Strain	Genotype	Reference
CEA17	*akuB (KU80)Δ*, *MAT1-1*	(1)
Δ*mpkA*	Δ*mpkA::pyrG, akuB (KU80)Δ*, *MAT1-1*	(2)
Δ*rlmA*	Δ*rlmA::pyrG, akuB (KU80)Δ*, *MAT1-1*	(3)
Δ*crzA*^{CEA17}	Δ*crzA*::*pyrG, akuB (KU80)Δ*, *MAT1-1*	(4)
CrzA::GFP	*CrzA::GFP::pyrG, akuB (KU80)Δ*, *MAT1-1*	(4)
Δ*crzA*::*crzA*$^{+}$	Δ*crzA*::*crzA*$^{+}$::*pyrG, akuB (KU80)Δ*, *MAT1-1*	(4)
Δ*zipD*	Δ*zipD*::*pyrG, akuB (KU80)Δ*, *MAT1-1*	This work
Δ*zipD*::*zipD*$^{+}$	Δ*zipD*::*zipD*$^{+}$::*prtA, akuB (KU80)Δ*, *MAT1-1*	This work
ZipD::GFP	*zipD*::*GFP*::*pyrG, akuB (KU80)Δ*, *MAT1-1*	This work

References

1. **Da Silva Ferreira ME, Kress MR, Savoldi M, Goldman MH, Härtl A, Heinekamp T, Brakhage AA, Goldman GH.** 2006. The *akuB*(KU80) mutant deficient for nonhomologous end joining is a powerful tool for analysing pathogenicity in *Aspergillus fumigatus*. Eukaryot Cell **5**:207-211.

2. **Valiante V, Heinekamp T, Jain R, Härtl A, Brakhage AA.** 2008. The mitogen-activated protein kinase MpkA of *Aspergillus fumigatus* regulates cell wall signaling and oxidative stress response. Fungal Genet Biol **45**:618-627.

3. **Rocha MC, Fabri JH, Franco de Godoy K, Alves de Castro P, Hori JI, Ferreira da Cunha A, Arentshorst M, Ram AF, van den Hondel CA, Goldman GH, Malavazi I.** 2016. *Aspergillus fumigatus* MADS-Box Transcription Factor RlmA Is Required for Regulation of the Cell Wall Integrity and Virulence. G3 **6**:2983-3002.

4. **Soriani FM, Malavazi I, da Silva Ferreira ME, Savoldi M, Von Zeska Kress MR, de Souza Goldman MH, Loss O, Bignell E, Goldman GH**. 2008. Functional characterization of the *Aspergillus fumigatus* CRZ1 homologue, CrzA. Mol Microbiol **67:**1274-1291.
