# Supplementary material for: The Aspergillus fumigatus CrzA Transcription Factor Activates Chitin Synthase Gene Expression during the Caspofungin Paradoxical Effect
Source: mBio. 2017 Jun 13;8(3):e00705-17. doi: 10.1128/mBio.00705-17 (PMC5472186; doi:10.1128/mBio.00705-17)
Supplement: TABLE S3 [file mbo003173337st3.pdf]

Primer name	Sequence	ORF number
chsA-F	5’-CCGTAGACTAATTGGATG-3’	Afu2g01870 (chsA)
chsA-F	5’-GGCTGAGAACGGAGTAGAAA-3’	Afu2g01870 (chsA)
csmB-F	5’-CTTTCCTATTCCACGCTTG-3’	Afu2g13430 (csmB)
csmB-R	5’-CCCTTGGTGTTGAGCGAAG-3’	Afu2g13430 (csmB)
chsC-F	5’-GTGGCGGAGCCCGCAAAG-3’	Afu5g00760 (chsC)
chsC-R	5’-GAACAATCGTCAGCCAAG-3’	Afu5g00760 (chsC)
chsG-F	5’-GCCTCATTTGTATACCTAAG-3’	Afu3g14420 (chsG)
chsG-R	5’-GAAAAAAGAAAAATTACTG-3’	Afu3g14420 (chsG)
mchsA-F	5’-CTAAAATCCAAGAAGTGACTGCTTGTAAGCCTGCCAAGCAAAG-3’	Afu2g01870 (chsA)
mchsA-R	5’-CTTTGCTTGGCAGGCTTACAAGCAGTCACTTCTTGGATTTTAG-3’	Afu2g01870 (chsA)
mcsmB-F	5’-CCGTTTCCATTCGTCTGATAGTAAGGTTGCCAATCAAACG-3’	Afu2g13430 (csmB)
mcsmB-R	5’-CGTTTGATTGGCAACCTTACTATCAGACGAATGGAAACGG-3’	Afu2g13430 (csmB)
mchsC-F	5’-GCCTTACAACGGGTGTCGCTGATTGTTGGCGGCGACAACCC-3’	Afu5g00760 (chsC)
mchsC-R	5’-GGGTTGTCGCCGCCAACAATCAGCGACACCCGTTGTAAGGC-3’	Afu5g00760 (chsC)
mchsG-F	5’-AACATTGCCTGGCGGCTCTGCTTGTGTTTCTTCCTTACTG-3’	Afu3g14420 (chsG)
mchsG-R	5’-CAGTAAGGAAGAAACACAAGCAGAGCCGCCAGGCAATGTT-3’	Afu3g14420 (chsG)
chsA Fw	5’-CGTCATCAAAGTATTATTTCCCC-3’	Afu2g01870 (chsA)
chsA Rv	5’-TTGGCTGAGAACGGAGTA-3’	Afu2g01870 (chsA)
chsC Fw	5’-ACGTTTAGACCACACCAAA-3’	Afu5g00760 (chsC)
chsC Rv	5’-GCCAAGAACCAACCAGT-3’	Afu5g00760 (chsC)
chsG Fw	5’-GTGGTTCGTGGGCTT-3’	Afu3g14420 (chsG)
chsG Rv	5’-GAAAGGCTGGTCGAGG-3’	Afu3g14420 (chsG)
csmB Fw	5’-TCTTTCGGCTTGTAGAAGTT-3’	Afu2g13430 (csmB)
csmB Rv	5’-ATTCTCTTCCCTTGGTGTTG-3’	Afu2g13430 (csmB)
